# A Synergistic Inhibitor Development Strategy Against Human UDP‐Galactose‐4‐Epimerase

**DOI:** 10.1002/anie.202520304

**Published:** 2026-04-20

**Authors:** William M. Browne, Jonathan Pettinger, Teresa Weckwerth, Andrew Purkiss, Sing Hei Lok, Louisa Penicaut, Roksana Ogrodowicz, Raveena Prema, Simone Kunzelmann, Chloe Roustan, Ganka Bineva‐Todd, Saskia Pieters, Francesca Zappacosta, Alfred E. Doherty, Isobel Oram, Christelle Soudy, Robert Quinlan, Joanna Redmond, Svend Kjaer, David House, Stephane Mouilleron, Jacob T. Bush, Benjamin Schumann

**Affiliations:** ^1^ Chemical Glycobiology Laboratory Francis Crick Institute London UK; ^2^ Department of Chemistry Imperial College London London UK; ^3^ GSK, Gunnels Wood Road Stevenage UK; ^4^ Structural Biology Science Technology Platform Francis Crick Institute London UK; ^5^ GSK, South Collegeville Road Collegeville USA; ^6^ Pure and Applied Chemistry University of Strathclyde Glasgow UK; ^7^ Chemical Biology Science Technology Platform Francis Crick Institute London UK; ^8^ Faculty of Chemistry and Food Chemistry TUD Dresden University of Technology Dresden Germany

**Keywords:** covalent, drug discovery, fragment‐based, glycosylation, metabolism

## Abstract

O‐GalNAc (*N*‐acetylgalactosaminyl) glycosylation is an abundant posttranslational modification in mammalian cells. Dysregulation of O‐GalNAc glycosylation is implicated in cancer metastasis and immune evasion; however, our mechanistic understanding remains limited due to the lack of small‐molecule tools. O‐GalNAc biosynthesis depends heavily on the availability of UDP‐GalNAc that is biosynthesised by the cytosolic enzyme UDP‐galactose‐4‐epimerase (GalE). Knockout studies have demonstrated that loss of GalE severely impairs O‐GalNAc glycosylation, positioning GalE as a promising enzymatic therapeutic target in oncology. Here, we present an efficient workflow that combines both covalent and high‐throughput crystallographic non‐covalent fragment screening with structure‐based design to identify GalE inhibitors. Using these strategies, we discovered a ligandable pocket adjacent to a reactive tyrosine, enabling the development of a potent, “beyond cysteine” sulfonyl fluoride covalent inhibitor as well as a derived covalent alkyne probe. Structurally‐enabled fragment screening methodologies yielded nanomolar non‐covalent as well as covalent binders within no more than 22 elaborated compounds. Our work demonstrates synergism in next‐generation delivery of chemical matter for GalE inhibition, with the broader potential for targeting non‐cysteine residues in chemical biology and therapeutic applications.

## Graphical abstract

The epimerase GalE is crucial for the biosynthesis of cancer‐relevant O‐GalNAc glycans. Here, we employ orthogonal, structurally enabled small molecule fragment screens to yield both covalent and non‐covalent inhibitors against GalE within no more than 22 elaborated compounds.

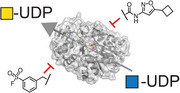

## 1 Introduction

Glycosylation with *N*‐acetylgalactosamine (GalNAc) is an abundant posttranslational modification in all mammalian cells [1]. Characterised by a glycosidic linkage between a serine or threonine residue and the sugar GalNAc, so‐called O‐GalNAc glycosylation is established in the secretory pathway [2]. Glycosyltransferases elaborate the core GalNAc to a host of more complex structures [3]. In human biology, O‐GalNAc glycans are critical for numerous physiological processes, including protein folding, regulation, and immune recognition [4]. Aberrant O‐GalNAc glycosylation in the form of shortened glycans and altered glycosylation patterns is heavily implicated in the pathogenesis of various cancers, contributing to metastasis and immune evasion [5, 6]. Notably, densely O‐GalNAc glycosylated mucin proteins serve as diagnostic markers for breast (MUC1) and ovarian cancer (MUC16) [7, 8]. Despite their significance, the precise mechanisms through which mucins, and more broadly, O‐GalNAc glycans, function in both normal and malignant cells remain poorly defined, and their investigation suffers from a shortage of chemical tools [9, 10, 11]. For example, while studying similarly abundant Asn(N)‐linked glycosylation benefits from the availability of small molecule inhibitors that selectively prevent specific elaboration in the N‐glycan biosynthetic pathway, no inhibitors are known to prevent O‐GalNAc glycosylation [12].

Chemical tools that further our understanding of O‐GalNAc glycosylation are critical, ideally in the form of a target‐selective yet functionally universal inhibitor. Protein O‐GalNAc glycosylation is highly redundant, with a family of up to 20 GalNAc transferase isoenzymes adding the first GalNAc moiety to Ser/Thr [2, 13]. Due to this redundancy, the process of glycosylation itself is unlikely to be universally druggable, albeit inhibitors against individual isoenzymes are highly interesting for specific applications [14, 15, 16].

**FIGURE 1:**
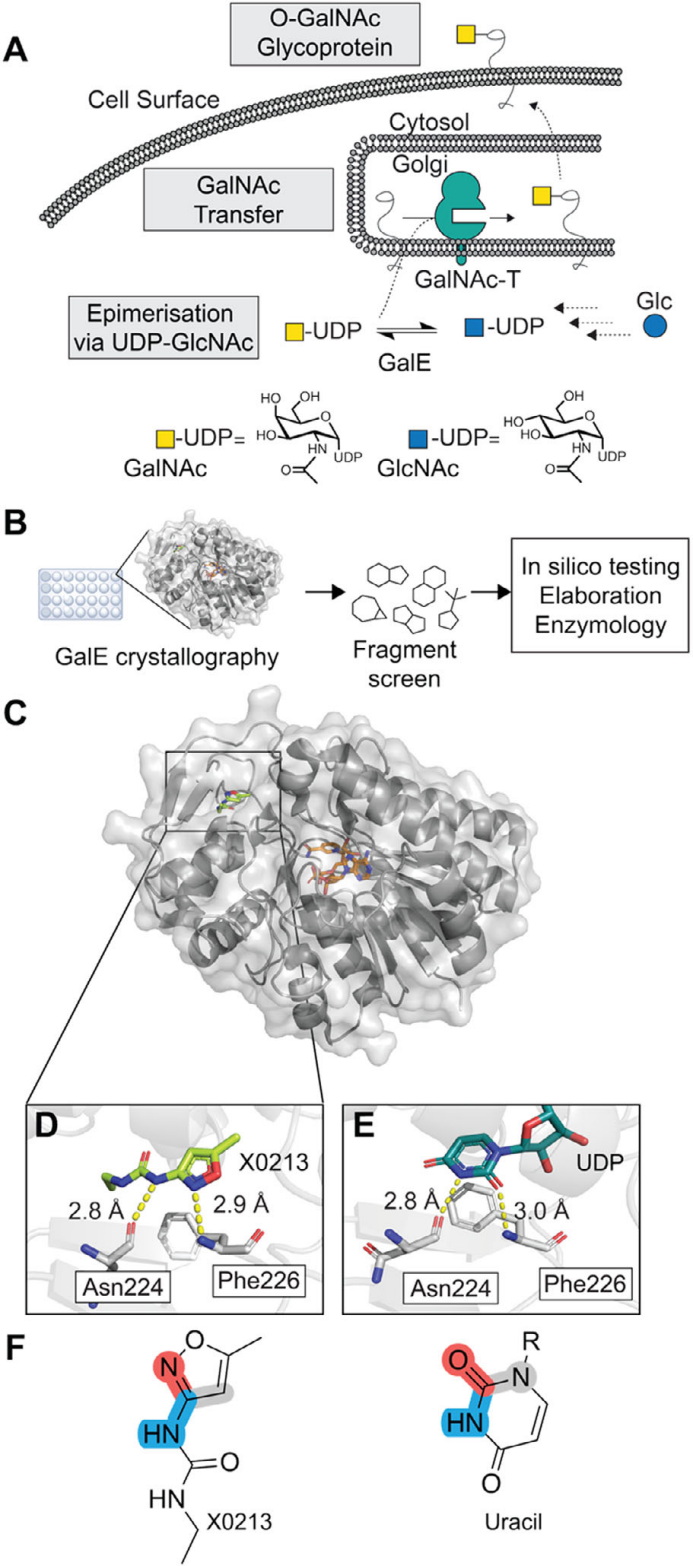
(A) Illustration showing the biosynthesis of an O‐GalNAc glycosylated protein with the key steps noted. (B) Outline of fragment screening and elaboration workflow. (C) Ligand bound X‐ray structure of hit X0213 (light green) in the substrate binding pocket of GalE monomer A (PDB: 9HJN). (D) Enhanced view of X0213 showing key hydrogen bonding (yellow dashed) to residues Asn224 and Phe226. (E) X‐ray structure of GalE in complex with UDP‐Glc (PDB: 1EK6) showing key hydrogen bonding partners of the UDP moiety. (F) Illustration highlighting X0213 acting as an isostere for the uracil moiety of UDP‐Glc. All structures displayed using Pymol v3.1.6.1 (Schrödinger).

All cellular O‐GalNAc glycan biosynthesis begins with the activity of glycosyltransferases that use the activated precursor uridine diphosphate (UDP)‐GalNAc [2]. As a cellular commodity that is primarily used for this purpose, biosynthesis of UDP‐GalNAc is an important bottleneck in O‐GalNAc glycosylation. Two distinct metabolic routes are known to produce UDP‐GalNAc. Free cellular GalNAc is recycled by enzymes of the salvage pathway that generate UDP‐GalNAc through sequential anomeric phosphorylation and condensation with uridine triphosphate (UTP) [17, 18]. In contrast, the major biosynthetic pathway features epimerisation of the C4‐epimer of UDP‐GalNAc termed UDP‐*N*‐acetylglucosamine (GlcNAc) by the enzyme UDP‐galactose‐4‐epimerase, GalE (Figure 1A) [19]. GalE is a cytosolic enzyme that also performs the analogous reaction to epimerize UDP‐galactose (Gal) and UDP‐glucose (Glc) [19, 20, 21]. Both functions are unique to GalE and proceed via hydride abstraction from the C4 position by the cofactor NAD (nicotinamide adenine dinucleotide) and subsequent reinsertion on the C4 of the inverted hexose ring [22, 23].

Previous studies knocked out *GALE* in CHO, K562, HEK, and HeLa cell lines and demonstrated a near total lack of O‐GalNAc glycosylation due to UDP‐GalNAc depletion that could not be rescued by the free GalNAc available in serum‐containing media [24, 25, 26, 27, 28]. Thus, GalE represents an attractive enzymatic target to curtail O‐GalNAc glycosylation by inhibiting UDP‐GalNAc biosynthesis. Two previous attempts have targeted GalE inhibition [29, 30]. A uridine analogue named 1–143 was developed by Winans and Bertozzi. However, the therapeutic or probe development potential of this class of compounds is limited by several factors, including the high molecular weight, high lipophilicity, and poor aqueous solubility. Urbaniak and Ferguson screened a set of small natural and commercial molecules to identify inhibitors selective for *Trypanosoma brucei* GalE over human GalE. While promising, some identified compounds were already known to target other therapeutic pathways, and some exhibited high intrinsic reactivity. Both studies elegantly demonstrated the tractability of GalE towards small molecule inhibition, but were limited by potency, reactivity, or permeability due to the chemical space available at the time.

We reasoned that emerging chemical biology strategies for ligand identification and optimization could expedite the discovery of a GalE inhibitor. Recent years have seen rapid progress in fragment screening approaches, high‐throughput crystallography, virtual docking, and the use of covalent warheads [31]. The rational discovery of targeted covalent inhibitors has regained significant interest in industry due to their potential for enhanced potency, prolonged duration of action, and improved selectivity, but has historically been restricted to targeting cysteine [32, 33, 34, 35, 36]. This limits the scope since many clinically relevant proteins lack exposed cysteines. Alternative ‘beyond cysteine’ warheads are gaining importance, with sulfur(VI) based warheads being highly versatile. For example, reaction with the phenolic hydroxyl group of tyrosines (or other potentially nucleophilic amino acid groups) with the sulfonyl fluoride warhead triggers the so‐called sulfonyl fluoride exchange (SuFEx) reaction that generates stable sulfonate esters [37, 38]. The reaction is particularly attractive due to the tunability through adjacent functional groups, suggesting a delicate balance between reactivity and stability towards non‐specific reaction or hydrolysis. *Meta*‐substituted, aromatic sulfonyl fluorides have been found to be particularly attractive for the development of Tyr‐reactive covalent probes [37, 38]. Despite the specificity and flexibility of SuFEx chemistry, broader adoption of non‐cysteine covalent warheads is still needed in the development of cellular tools and clinical candidates.

Herein, we establish a workflow for covalent inhibitor discovery that integrates orthogonal fragment screening, ultra‐high resolution structural biology, and docking for GalE as a clinically relevant target. Structure‐based non‐covalent fragment screening and elaboration expedite the definition of a ligandable pocket in GalE. Identification of a nearby reactive tyrosine enables the creation of a sulfonyl fluoride‐containing compound as a first‐in‐kind potent and ‘beyond‐cysteine’ covalent GalE inhibitor to a clinically relevant target, with further application as a bioorthogonal covalent probe.

## 2 Results and Discussion

Key to inhibitor discovery is the identification of a ligandable pocket that leads to functional modulation of the protein. We reasoned that the binding pocket for the abundant cofactor NAD was unsuitable based on the likely tight affinity to GalE. In contrast, the UDP‐sugar binding site offered an attractive target. A high‐throughput crystallographic screen of non‐covalent fragments was performed to identify pharmacophores for this site on recombinant human GalE (Figure 1B).

After identifying reproducible crystallography conditions, a poised (DSI) fragment library of 857 non‐covalent compounds was screened by crystal soaking employing automated dispensing in ethylene glycol (EG) [19, 22]. A pilot screen was first conducted with 250 fragments at 20 mM concentration (20% EG). Since these conditions appeared to cause crystal fracturing, the remaining 607 compounds were soaked at 10 mM concentration (10% EG) to maintain crystal integrity. The 20 mM pilot screening dataset identified an isoxazole X0213 in the substrate pocket of GalE. Fragment X0213 was re‐soaked into GalE crystals to obtain a higher quality data set and confirm GalE binding (Figure 1C,D, PDB 9HJN). Notably, the obtained structure revealed that the urea moiety of X0213 formed hydrogen bonding interactions to Asn224 and Phe226, analogous to the interactions formed by the uracil moiety of UDP‐sugars (Figure 1E,F). Protein binding was subsequently quantified by isothermal titration calorimetry (ITC), revealing an approximate *K_D_* for isoxazole X0213 of 468 ± 127 µM (Figure S1A).

**FIGURE 2:**
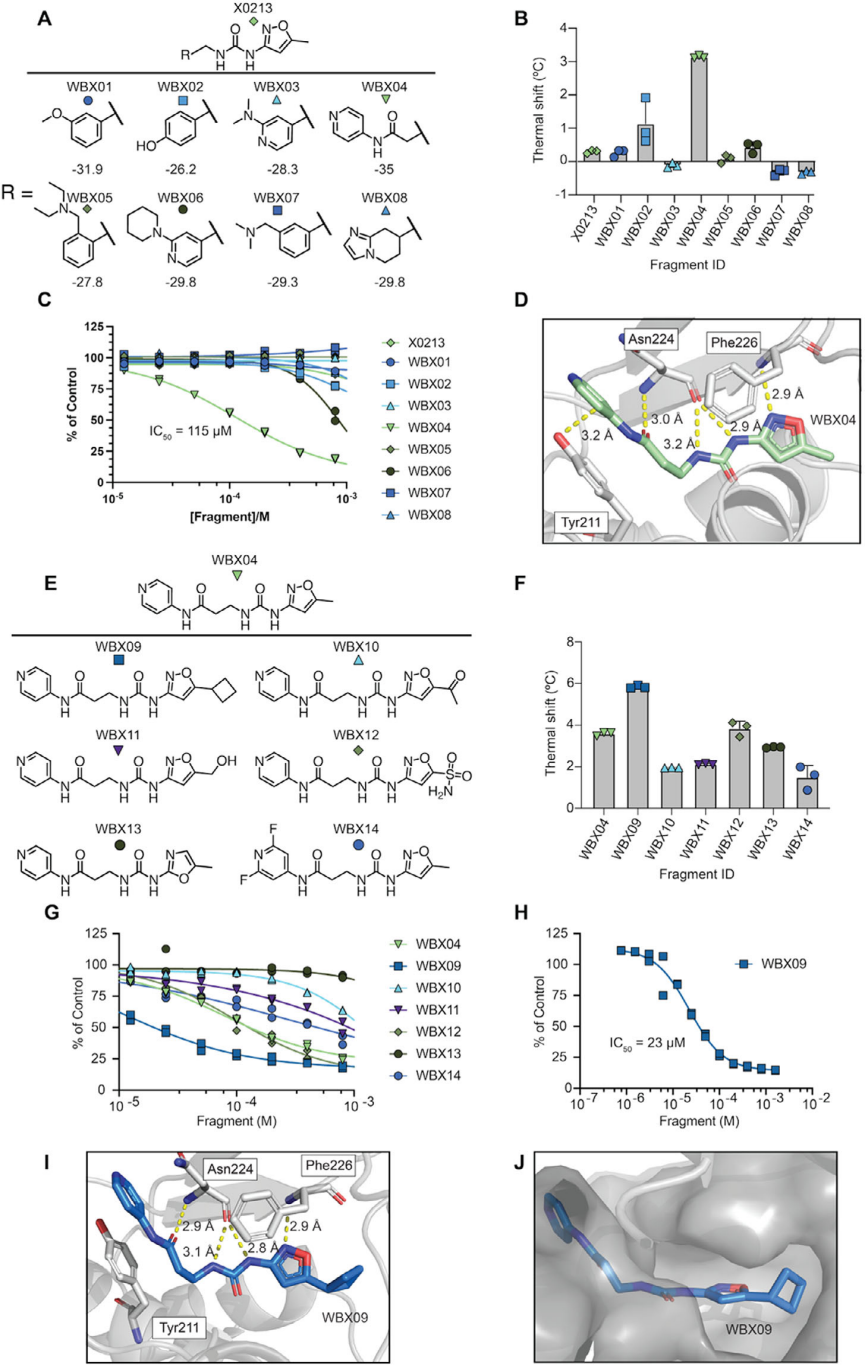
(A) Structures of the first series of non‐covalent analogues (WBX01‐WBX08) with ICM docking score. (B) DSF thermal shift assay Δ*T*_m_ for hit X0213 and analogues WBX01‐WBX08. Data are means + SD of three technical replicates relative to the mean of three DMSO controls. (C) Concentration‐response biochemical assay results for hit X0213 and analogues WBX01‐WBX08. Data are individual values from two technical replicates. (D) X‐ray structure (PDB: 9HI0) of WBX04 (pale green) bound to GalE with hydrogen bonding (yellow dashed) to Asn224 and stacking interaction with Tyr211. (E) Structures of the second series of non‐covalent analogues (WBX09‐WBX14). (F) DSF assay Δ*T*_m_ for compounds WBX09‐WBX14. Data are means + SD of three technical replicates relative to the mean of three DMSO controls (G) Concentration‐response biochemical assay results for WBX09‐WBX14. Data are individual values from two technical replicates. (H) Extended concentration response curve for fragment WBX09. Data are individual values from two technical replicates. (I) X‐ray structure (PDB: 9HI1) of WBX09 (blue) in complex with GalE with hydrogen bonding interactions (yellow dashed). (J) WBX09 in complex with GalE with Pymol modelled surface showing accommodation of the cyclobutyl group in a pocket adjacent to the isoxazole ring. All curves were fitted using Graphpad Prism (v10.3.0) non‐linear curve fitting. All structures were displayed using Pymol v3.1.6.1 (Schrödinger).

We next aimed to elaborate X0213 to a more potent binder that would effectively inhibit GalE. A total of 1404 commercially available analogues of X0213 were docked into GalE (using the X0213‐bound structure), maintaining the structurally important methyl isoxazole moiety in X0213 while seeking additional compound‐protein interactions. Analogues were ranked using the ICM scoring function, and eight compounds (WBX01 to WBX08, Figure 2A) were selected for characterisation based on their ability to form additional H‐bonding and π‐stacking interactions that we rationalised may yield an increased affinity for GalE. These analogues were characterised with a workflow of differential scanning fluorimetry (DSF), GalE enzyme inhibition, and, for the most active compounds, ITC (Figure S2). The GalE enzyme assay uses high‐performance anion exchange chromatography (HPAEC) to determine the conversion of UDP‐Gal to UDP‐Glc (Figure S3A) [28]. Fragment WBX04, displaying a terminal pyridyl amide, was a substantially tighter binder than the parent compound X0213, with a Δ*T*_m_ of 3.1°C (Figure 2B) and inhibition of enzyme activity with an IC_50_ of 115 µM (Figure 2C). WBX04 displayed an estimated K_D_ of 9 ± 6 µM by ITC (Figure S1B), an approximately 100‐fold increase in affinity over the parent compound X0213. We obtained a crystal structure for WBX04 soaked in the active site of GalE at 1.4 Å resolution (Figure 2D). Structural analysis revealed that the affinity of WBX04 to GalE was likely the result of hydrogen bonds to the backbone of residue Asn224 at 2.9, 3.0 and 3.2 Å, respectively, as well as a favourable π‐stacking interaction with Tyr211 (Figure 2D). The distance of the Tyr211 hydroxyl group to the closest carbon atom in the pyridyl ring was measured as 3.2 Å.

We next aimed to further improve compound potency with a second series of WBX04 analogues (Figure 2E). Compounds WBX09 to WBX12 included substitutions of the terminal methyl isoxazolyl group with bulkier functionalities, driven by the availability of additional space at that position in GalE. WBX13 constituted a pyridyl regioisomer, while WBX14 reduced the ring electron density. Among these compounds, cyclobutyl‐displaying WBX09 presented another major step in compound optimization, exhibiting improved thermal stabilisation (Δ*T*_m_ 5.8°C, Figure 2F) and enzyme inhibition (IC_50_ 23 µM, Figure 2G,H), and a further 18‐fold increase in affinity (*K*_D_ ∼ 500 nM, Figure S1C) compared to WBX04. Next, we assessed the selectivity of WBX09 towards enzymes that accept structurally related substrates. UDP‐*N*‐acetylhexosamine pyrophosphorylase (AGX1) converts GalNAc‐1‐phosphate to UDP‐GalNAc, whilst 1,6‐mannosyl‐glycoprotein‐2‐*N*‐acetylglucosaminyltransferase (MGAT2) transfers a GlcNAc monosaccharide from UDP‐GlcNAc to an N‐linked glycan precursor [39, 40]. As GalE accepts both UDP‐GalNAc and UDP‐GlcNAc, a promiscuous inhibitor might be expected to affect both enzymes. We therefore assessed the effect of high concentrations of WBX09 (400 µM, > 17‐fold the GalE IC_50_) on both MGAT2 and AGX1 activity. No inhibition was observed across multiple enzyme concentrations (Figure S3B,C), confirming the extent to which both enzymes remained unaffected, even under high substrate conversion conditions where subtle effects on activity would be observed.

We obtained a GalE crystal structure binding WBX09 at 0.95 Å. Conserving interactions with Tyr211, Asn224 and Phe226 (Figure 2I), structural analysis suggested that the increase in potency was driven by filling a pocket lined by hydrophobic Tyr230 with the cyclobutyl group (Figures 2J and S4). We note that further optimization will be needed to enable cellular inhibitor activity, as a preliminary DMPK revealed that WBX09 has a hepatocyte half‐life of 16.4 min and an intrinsic clearance of 42.4 µL/min/million cells. Permeability was measured in an MDCK monolayer assay, showing an efflux ratio of 35.8 and suggesting that WBX09 is likely a P‐glycoprotein substrate. Taken together, structure‐based optimization successfully elaborated an initial fragment hit to a high nanomolar binder of GalE with as few as 14 synthetic compounds.

**FIGURE 3:**
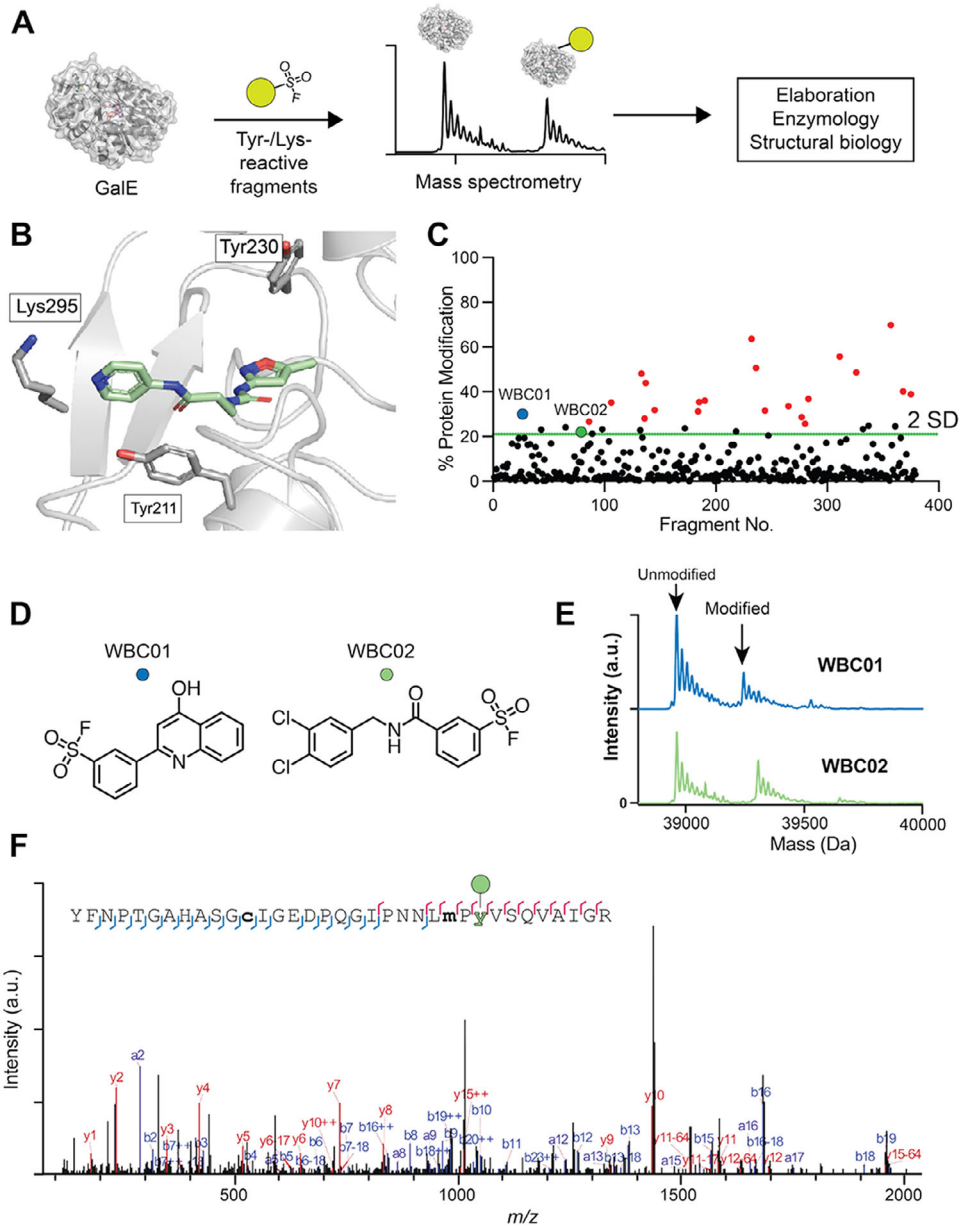
(A) Outline of covalent fragment screening and elaboration workflow. (B) X‐ray structure of WBX04 in complex with GalE showing nucleophilic side chain residues (grey). (C) Sulfonyl fluoride library intact‐protein LCMS screening results, showing fragment number versus ratio (%) of GalE modification. Hit line represents two standard deviations from the mean. Screening was performed as one experiment. Hits WBC01 and WBC02 are shown in blue and green, respectively. Compounds that produced multiple modification adducts shown in red. (D) Chemical structures of the covalent screening hits WBC01 and WBC02. (E) Deconvoluted mass spectrum for fragments WBC01 (blue) and WBC02 (green) from intact‐protein LCMS screening. Data are from one experiment. (F) Representative tryptic digest LCMS/MS spectra for a Tyr211 containing peptide modified by fragment WBC02. b ions are shown in blue and y ions in red. Lower case denotes modification (oxidation for methionine and cysteine). Green sphere signifies covalent adduct. Data are from one experiment. Spectra generated in Byonic (ProteinMetrics, v4.0.12).

To advance GalE inhibitors towards a suitable probe, we next evaluated available crystal structures to identify nearby nucleophilic residues for developing covalent inhibitors (Figure 3A). Three residues close to the ligand binding pocket were identified as potential modification points for commonly employed covalent warheads, Tyr211, Tyr230 and Lys295 (Figure 3B). We screened a library of 347 sulfonyl fluoride‐containing fragments, reported to target Tyr/Lys residues (Figure 3C). Screening was performed by incubating fragments at 100 µM with 0.5 µM purified GalE for 24 h. Protein modification was then determined by intact‐protein LCMS. In this experiment, we deliberately omitted the addition of the NAD cofactor to keep the experimental conditions simple and potentially allow for the sampling of a larger conformational ensemble in GalE.

The screen yielded 12 fragments with a suitable hit profile of more than 21% of GalE being modified, representing two standard deviations from the mean, and single protein modification (Figure 3C). Two hits, WBC01 and WBC02, displayed the most suitable reactivity profiles with mono‐adducts or very minor di‐adducts (Figure 3D,E). Tryptic digest mass spectrometry (MS) analysis of GalE incubated with WBC01 or WBC02 at either 100, 50 or 25 µM concentrations unambiguously verified Tyr211 as the primary target residue modified by both compounds (Figures 3F and S5). These data were supported by previous annotation of Tyr211 as a reactive residue by proteome‐wide profiling with sulfonyl triazoles [41]. Thus, Tyr211 appeared to be a promising target residue for the development of a covalent inhibitor from our existing non‐covalent binders.

**FIGURE 4:**
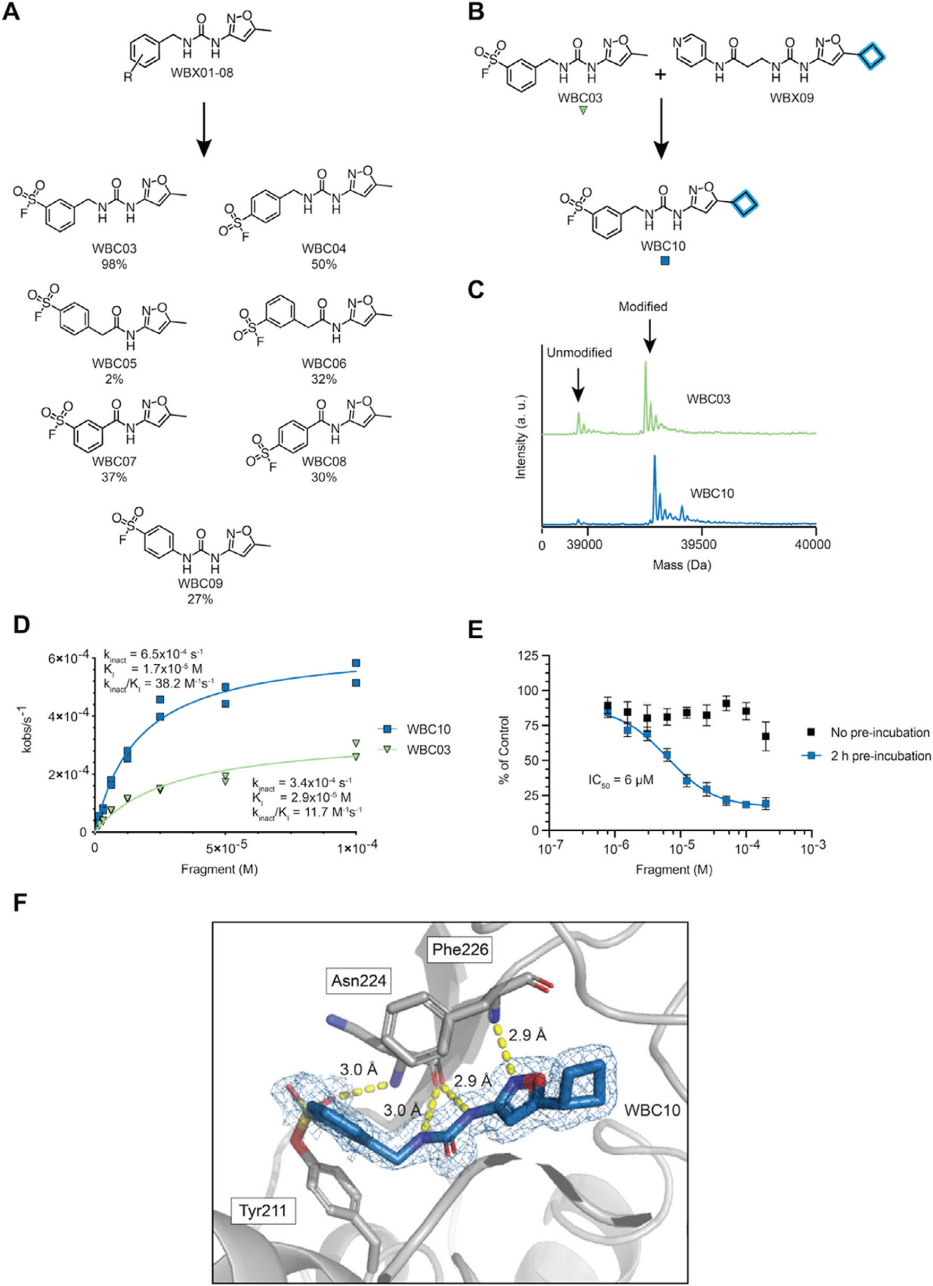
(A) Chemical structures and activity of covalent WBC compound series. Protein modification % after incubation of 0.5 µM GalE with 100 µM compound by intact‐protein LCMS shown below analogue. Data are from one experiment. (B) Illustration showing the rational design of compound WBC10. (C) Intact‐protein LCMS spectra of WBC03 and WBC10 assessed at 6.25 µM fragment, 0.5 µM GalE, 5 h incubation. Data are from one experiment. (D) Intact‐protein LCMS kinetics experiment for WBC03 and WBC10. Experiment run with 100 to 1.56 µM fragment, 0.5 µM GalE. Data are from two independent replicates with each replicate plotted. (E) Concentration‐response curve for GalE with or without a 2‐h preincubation with 25 nM GalE and WBC10 at varying concentrations. Protein‐compound incubations were run in three independent replicates with two technical replicates each. Data are means ± SEM. (F) 1.4 Å crystal structure (PDB: 9HI2) of compound WBC10 (blue) with covalent modification of Tyr211 (grey) with key hydrogen bonding interactions (yellow) to Asn224 and Phe226 (grey) and 2Fo‐Fc 1.0 σ density map for WBC10 and Tyr211 (blue).

Through orthogonal fragment screening, we defined a tractable pocket for GalE inhibition in close proximity to a nucleophilic Tyr residue. To evolve non‐covalent into covalent inhibitors, we designed seven compounds based on our WBX series (WBC03‐09) bearing a Tyr‐reactive sulfonyl fluoride warhead and maintaining the essential isoxazole functional group (Figure 4A). In this initial series, WBX04 was used as an early structural starting point for covalent inhibitor design. Compounds were designed to probe both the effect of warhead orientation and the relevance of hydrogen bonding patterns on fragment binding.

Covalent compounds WBC03‐09 were screened by intact‐protein LCMS to determine protein modification (Figures 4A and S6A–G). Employing the same assay conditions as in our previous sulfonyl fluoride screen (0.5 µM protein, 100 µM fragment), *meta‐*substituted fragment WBC03 produced > 95% protein mono‐modification. No adducts of higher fragment‐protein stoichiometry were observed (Figure S6A). In general, the *meta‐*substituted analogues WBC03, WBC06, and WBC07 all produced more complete protein modification over the *para‐*analogues WBC04, WBC05, WBC08, and WBC09. Compounds WBC07 and WBC08 produced the most non‐specific reactivity, which likely results from enhanced reactivity due to direct substitution of the electron‐withdrawing amide (Figure S6E,F).

We next sought to incorporate the additional affinity provided by a cyclobutyl‐functionalised isoxazole in the design of WBC10 (Figure 4B). Under more stringent assay conditions (1 µM protein, 6.25 µM fragment), cyclobutyl‐containing WBC10 displayed more efficient covalent modification than WBC03, while retaining complete selectivity for mono‐modification (Figure 4C). *k*_inact_ and *K*_I_ were determined to quantify covalent binding kinetics. Parameters were derived from time‐ and concentration‐dependent covalent modification, with *k*_obs_ fitted to a Michaelis–Menten model under pseudo‐first‐order conditions (Figure S7A,B) [42]. The calculated *k*_inact_/*K*_I_ value for WBC10 (38.2 M^−1^s^−1^) was approximately 3.5‐fold higher than for WBC03 (11.7 M^−1^s^−1^), with equal contributions from *K*_I_ and *k*_inact_ (Figure 4D), demonstrating the effect of the cyclobutyl moiety in WBC10 to refine the suitability as a specific covalent ligand for human GalE.

We validated cyclobutyl‐containing WBC10 as a covalent inhibitor of human GalE by pre‐incubating the protein with varying concentrations of WBC10, followed by enzyme activity assessment (Figure 4E for WBC10 of < 90% purity, Figure S8 for re‐synthesized WBC10 of > 95% purity). Concentration‐dependent inhibition was observed under these conditions (IC_50_ of ∼6 µM), comparable to non‐covalent WBX09. Control samples treated with WBC10 without a pre‐incubation period showed little GalE inhibition, consistent with a covalent mode of action. Finally, we confirmed that both fragments WBC03 and WBC10 modify Tyr211 of human GalE. Site identification experiments using tryptic digest LCMS/MS confirmed that only Tyr211 was being modified by both covalent analogues, even when incubating GalE with a 200‐fold excess of compound (Figure S9A,B). To assess how WBC10 interacts with GalE, we solved the crystal structure of the covalent WBC10‐GalE adduct. GalE was pre‐incubated with WBC10 overnight prior to crystal growth. A continuous electron density was found linking the phenolic oxygen in Tyr211 with the sulfur in the WBC10 sulfonyl group at a distance of 1.5 Å. The structure delineated the formation of a sulfonate ester with a tetrahedral symmetry and an O‐S‐C bond angle of 104.5°. Since prolonged exposure to synchrotron radiation fragmented this bond, only initial diffraction data were taken to produce the WBC10‐GalE crystal structure (Figure 4F). The structure highlighted that even after covalent bond formation, the binding mode observed in X0213, WBX04, and WBX09 was maintained for WBC10. Key hydrogen bond (Asn224 and Phe226) and hydrophobic interactions were conserved. Interestingly, an additional hydrogen bond was also observed between the sulfonyl oxygen and Asn224. Taken together, our data underpin the use of Tyr‐reactive covalent inhibitors against GalE.

**FIGURE 5:**
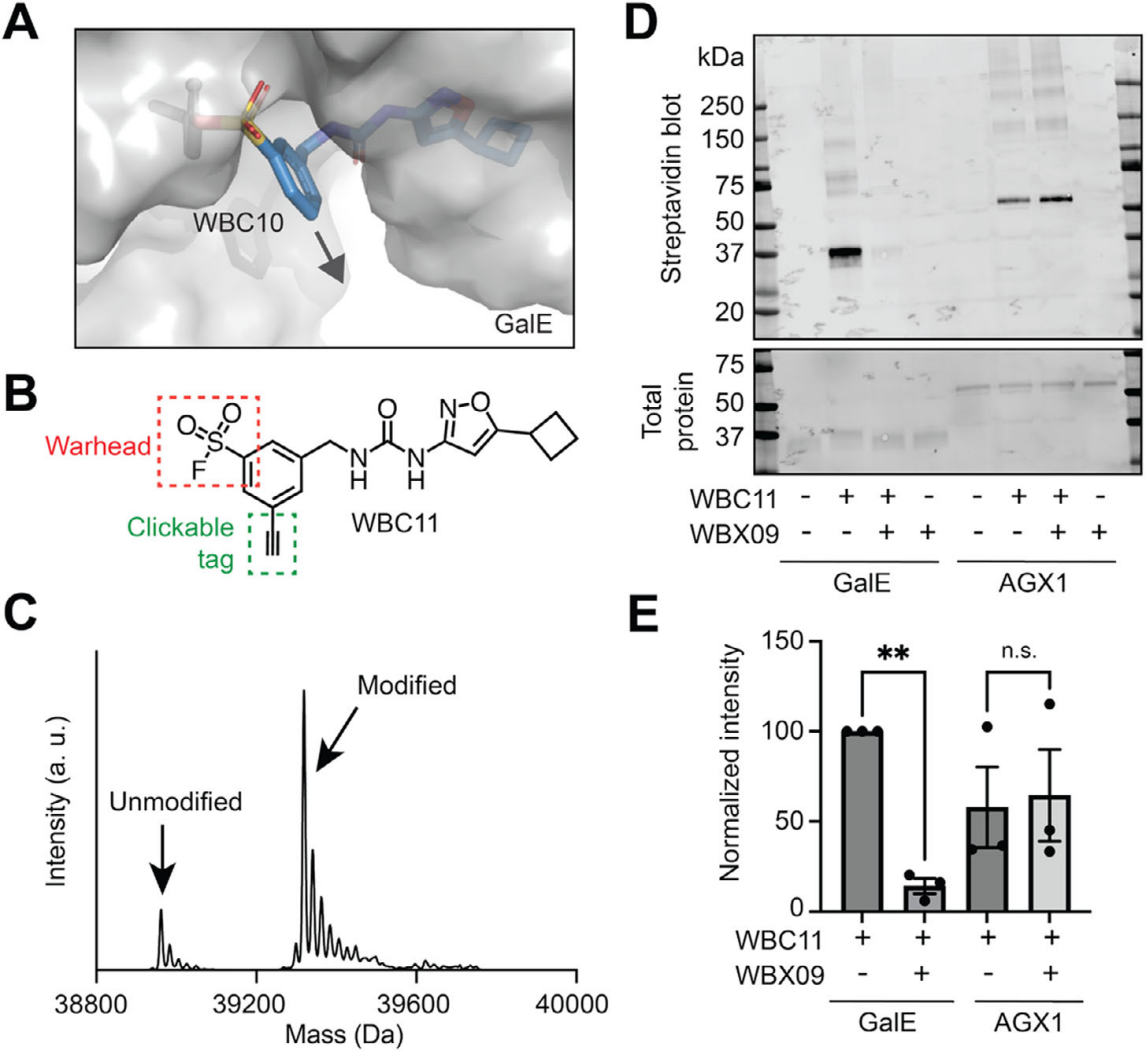
(A) Solvent accessibility of the phenyl ring in WBC10 shown in the crystal structure of the covalent adduct (PDB: 9HI2). All structures were displayed using Pymol v3.1.6.1 (Schrödinger). Arrow indicates vector of alkyne modification in WBC11. (B) Structure of clickable probe WBC11. (C) Intact‐protein LCMS spectra of WBC11 assessed at 5 µM fragment, 0.5 µM GalE. Data are from one experiment. (D) Streptavidin blot and total protein of 0.5 µg recombinant GalE or AGX1 treated with 10 µM WBC11 with or without 100 µM WBX09 as a competitor. (E) Quantification of normalized band intensities in (D). Data are means ± SEM from three independent experiments. Data were compared by two‐tailed, paired *t*‐test: **p < 0.01, n.s., not significant.

Covalent modifiers can be converted into traceable activity‐ or affinity‐based protein probes [43]. Having developed WBC10 as a suitable covalent inhibitor, we explored the conversion into a bioorthogonal derivative for direct visualization of GalE modification. Inspection of the GalE‐WBC10 co‐crystal structure revealed solvent exposure of the phenyl ring (Figure 5A). We appended an alkyne group to the structure of WBC10 toward the design of covalent, bioorthogonal modifier WBC11 (Figure 5B). Incubation of GalE with WBC11 confirmed similar single‐modification of GalE as the parental compound WBC10 (Figure 5C). We then used WBC11 to trace covalent GalE modification (Figure 5D). Recombinant GalE was incubated with 10 µM WBC11 in the presence or absence of a tenfold excess of WBX09 as a non‐covalent competitor. Subsequent copper‐catalysed azide‐alkyne cycloaddition (CuAAC) with biotin picolyl azide and streptavidin blot produced an intense band for GalE upon treatment with WBC11. Co‐incubation with WBX09 led to a near‐complete abrogation of chemical GalE tagging, consistent with competition of the covalent labelling reaction. Total protein staining suggested a visible shift in molecular weight only for GalE incubated with WBC11 without WBX09 co‐incubation. When the same treatment regimen was performed with AGX1 as a control protein, a biotin‐tagged band of lower intensity was observed that was not abrogated upon co‐incubation with WBX09. Treatment with WBX09 or DMSO did not lead to observable biotin tagging in all cases. The traceable nature of WBC11 allowed us to assess the stability of the sulfonyl ester bond to Tyr211. GalE treated with WBC11 was subjected to disruptive conditions at 95°C for 5 min in sodium dodecyl sulphate loading buffer. Subsequent CuAAC with biotin and streptavidin blot revealed conservation of the covalent bond, as the intensity of the GalE‐WBC11 adduct did not change after temperature increase (Figure S10). These data suggest that WBC11 is a traceable, affinity‐based covalent probe for GalE targeting Tyr211 as a modifiable site.

## 3 Conclusion

O‐GalNAc glycosylation is a complex and dynamic biological process with far‐reaching implications in human health. Here, we posit that GalE is a suitable target for small‐molecule intervention to eventually prevent O‐GalNAc glycosylation altogether. GalE is overexpressed in several prominent cancers according to expression data (Figure S11). Mutations in the *GALE* gene are subject to rare, inherited metabolic deficiencies that manifest in young children but tend to be less severe in adolescents and adults [44]. Nevertheless, we would anticipate global comprehensive inhibition of GalE to have effects on mucin‐heavy tissues such as the gastrointestinal tract. Hence, it will be important to establish a suitable therapeutic index in future inhibitor campaigns. Previous inhibitor discovery approaches have shown that GalE is druggable in principle but did not identify suitable chemical matter for chemical tool development [29, 30].

Here, our work focused on harnessing the information content provided by complementary fragment screens to allow for rapid optimization of both non‐covalent and covalent inhibitors. With advances in screening and medicinal chemistry revolutionizing drug discovery, our work showcases the power of structurally guided inhibitor optimisation in combination with ‘beyond‐cysteine’ covalent inhibition. We combined high‐throughput crystallographic fragment screening, biophysical and biochemical assays, high‐resolution structural insight, and computational docking to improve an initial fragment binder by ∼1000‐fold in potency to target the substrate binding pocket of GalE within no more than 14 elaborated intermediates. In parallel, we identified the residue Tyr211 close to the GalE active site as suitable for nucleophilic covalent modification through screening a library of aryl sulfonyl fluoride‐containing fragments. The increasing availability of high‐quality datasets depicting nucleophilic residues across the cellular proteome, combined with the latest advances in covalent ligand screening, will likely render this step even more efficient in the near future [45, 46, 47]. We identified a site‐specific covalent inhibitor of GalE by designing a compound that combined a sulfonyl fluoride warhead with the structural features of our non‐covalent binders. We then developed a covalent, bioorthogonal probe for Tyr211 in GalE. The use of sulfur(VI) reactive warheads is powerful but can be subject to non‐specific labelling background [37, 48, 49, 50]. Further development of covalent GalE‐targeting probes will therefore focus on the fine‐tuning of warhead reactivities, with progress being made in their proteomic evaluation [48]. Our work demonstrates the potential of combining state‐of‐the‐art workflows in screening, structural biology and computational modelling to provide rapid access to inhibitor compounds as highly valuable in vitro biochemical tools against a clinically relevant target.

## Conflicts of Interest

Jonathan Pettinger, David House and Jacob T. Bush are employees of GSK. The other authors declare no conflicts of interest.

## Supporting information




**Supporting File 1**: anie72084‐sup‐0001‐SuppMat.pdf.

## Data Availability

Deposition numbers 9HI0, 9HI1, 9HI2, and 9HJN contain the supplementary crystallographic data for this paper. These data are provided free of charge by the Protein Data Bank www.rcsb.org.
