# Supplementary material for: A Synergistic Inhibitor Development Strategy Against Human UDP‐Galactose‐4‐Epimerase
Source: Angew Chem Int Ed Engl. 2026 Apr 20;65(22):e20304. doi: 10.1002/anie.202520304 (PMC13206458; doi:10.1002/anie.202520304)
Supplement: Supplementary file 1 — Supporting File 1: anie72084‐sup‐0001‐SuppMat.pdf. [file ANIE-65-e20304-s001.pdf]

A Synergistic Inhibitor Development Strategy Against Human UDP-Galactose-4-Epimerase

Supporting Information

William M. Browne[a][b], Jonathan Pettinger[c], Teresa Weckwerth[a][b], Andrew Purkiss[d], Sing Hei Lok[a][b], Louisa Penicaut[a][b], Roksana Ogrodowicz[d], Raveena Prema[d], Simone Kunzelmann[d], Chloe Roustan[d], Ganka Bineva-Todd[a], Saskia Pieters[a][b], Francesca Zappacosta[e], Alfred E. Doherty[c][f], Isobel Oram[a][b], Christelle Soudy[g], Robert Quinlan[g], Joanna Redmond[g], Svend Kjaer[d], David House[c], Stephane Mouilleron[d], Jacob T. Bush[c], and Benjamin Schumann*[a][b][h]

[a] Chemical Glycobiology Laboratory, Francis Crick Institute, 1 Midland Road, London NW1 1AT, UK.

[b] Department of Chemistry, Imperial College London, 82 Wood Lane, London W12 0BZ, UK.

[c] GSK, Gunnels Wood Road, Stevenage, Hertfordshire, SG1 2NY, UK.

[d] Structural Biology Science Technology Platform, Francis Crick Institute, 1 Midland Rd, London NW1 1AT, UK.

[e] GSK, South Collegeville Road, Collegeville, PA 19426, USA.

[f] Pure and Applied Chemistry, University of Strathclyde, Thomas Graham Building, 295 Cathedral Street, Glasgow G1 1XL, UK.

[g] The Chemical Biology Science and Technology Platform, The Francis Crick Institute, 1 Midland Road, London NW1 1AT, UK

[h] Department of Chemistry and Food Chemistry, TUD Dresden University of Technology, 01069 Dresden, Germany.

* Correspondence: benjamin.schumann@tu-dresden.de

Table S1: Compounds ordered from Enamine with manuscript ID, Enamine catalogue ID and SMILES notation.

Manuscript ID	Enamine ID	SMILES
WBX01	Z166729656	COC1=CC=CC(=C1)CNC(=O)NC=2C=C(C)ON2
WBX02	Z285039432	CC1=CC(=NO1)NC(=O)NCC=2C=CC(O)=CC2
WBX03	Z374827866	CC1=CC(=NO1)NC(=O)NCC=2C=CN=C(C2)N(C)C
WBX04	Z992050546	CC1=CC(=NO1)NC(=O)NCCC(=O)NC=2C=CN=CC2
WBX05	Z166720732	CCN(CC)CC1=CC=CC=C1CNC(=O)NC=2C=C(C)ON2
WBX06	Z495790768	CC1=CC(=NO1)NC(=O)NCC=2C=CN=C(C2)N3CCCCC3
WBX07	Z362591558	CC1=CC(=NO1)NC(=O)NCC=2C=CC=C(C2)CN(C)C
WBX08	Z1756063853	O=C(NCC1CCN2C=CN=C2C1)NC=3C=CON3
WBX09	Z8301530801	O=C(CCNC(=O)NC=1C=C(ON1)C2CCC2)NC=3C=CN=CC3
WBX10	Z8301530848	CC(=O)C1=CC(=NO1)NC(=O)NCCC(=O)NC=2C=CN=CC2
WBX11	Z8301530783	O=C(CCNC(=O)NC=1C=C(CO)ON1)NC=2C=CN=CC2
WBX12	Z8301530837	NS(=O)(=O)C1=CC(=NO1)NC(=O)NCCC(=O)NC=2C=CN=CC2
WBX13	Z8301530862	CC1=CN=C(NC(=O)NCCC(=O)NC=2C=CN=CC2)O1
WBX14	Z8301530881	CC1=CC(=NO1)NC(=O)NCCC(=O)NC=2C=C(F)N=C(F)C2
WBC03	Z8309745310	CC1=CC(=NO1)NC(=O)NCC=2C=CC=C(C2)S(=O)(=O)F
WBC04	Z8687356596	CC1=CC(=NO1)NC(=O)NCC=2C=CC(=CC2)S(=O)(=O)F
WBC05	Z8309745299	CC1=CC(=NO1)NC(=O)CC=2C=CC(=CC2)S(=O)(=O)F
WBC06	Z8309745306	CC1=CC(=NO1)NC(=O)CC2=CC=CC(=C2)S(=O)(=O)F
WBC07	Z2759651909	CC1=CC(=NO1)NC(=O)C=2C=CC=C(C2)S(=O)(=O)F
WBC08	Z2759651889	CC1=CC(=NO1)NC(=O)C=2C=CC(=CC2)S(=O)(=O)F
WBC09	Z8309745319	CC1=CC(NC(NC2=CC=C(S(=O)(F)=O)C=C2)=O)=NO1
WBC10	Not in cat.	O=C(NCC1=CC(S(=O)(F)=O)=CC=C1)NC2=NOC(C3CCC3)=C2
WBC11	Not in cat.	O=C(NCC1=CC(S(=O)(F)=O)=CC(C#C)=C1)NC2=NOC(C3CCC3)=C2

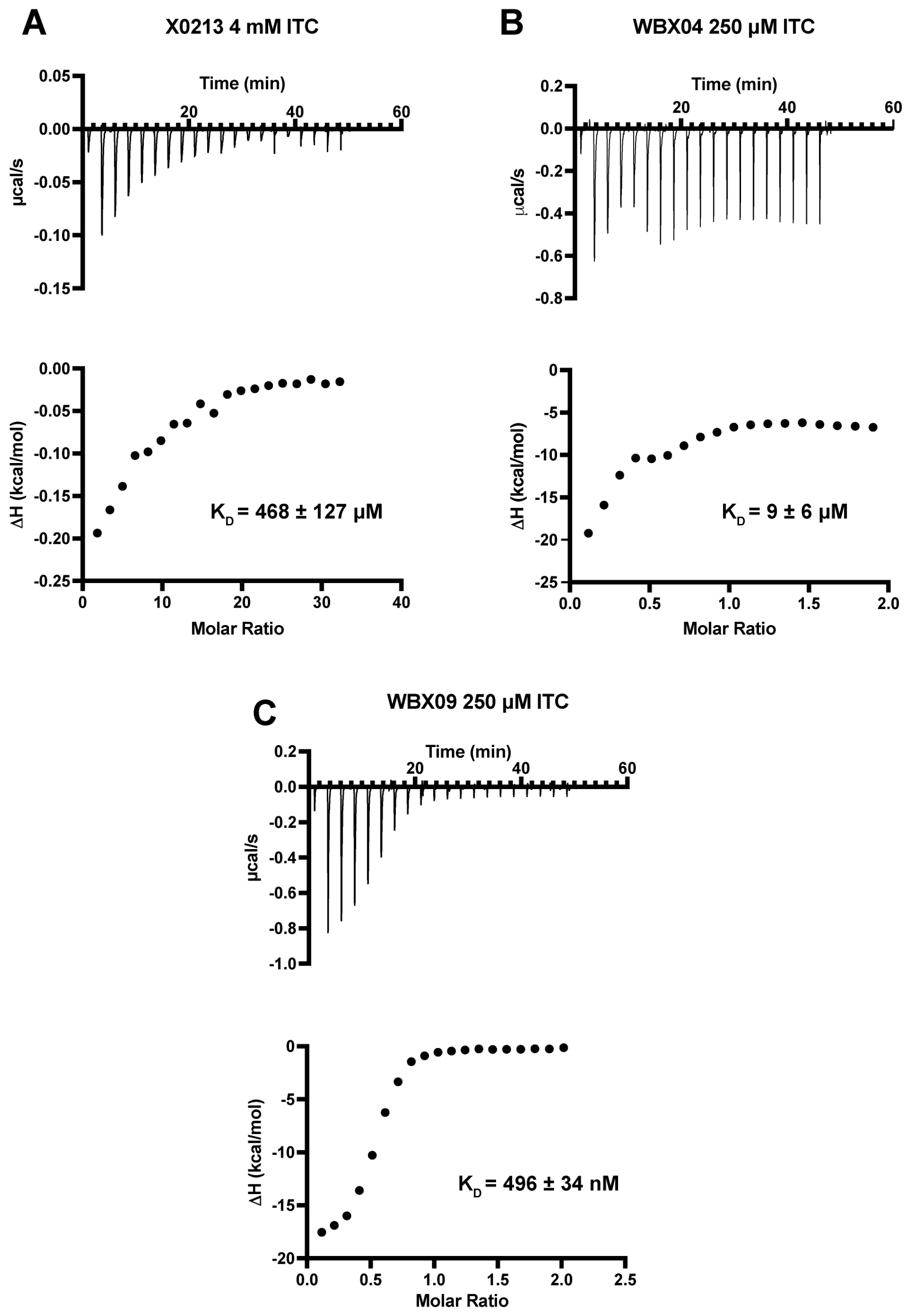

Fig. S1: ITC thermograms and integral plots for compounds X0213 (A), WBX04 (B) and WBX09 (C) with K_D. Integration analysis, curve fitting and error performed in MicroCal PEAQ ITC software. Data are from one experiment.

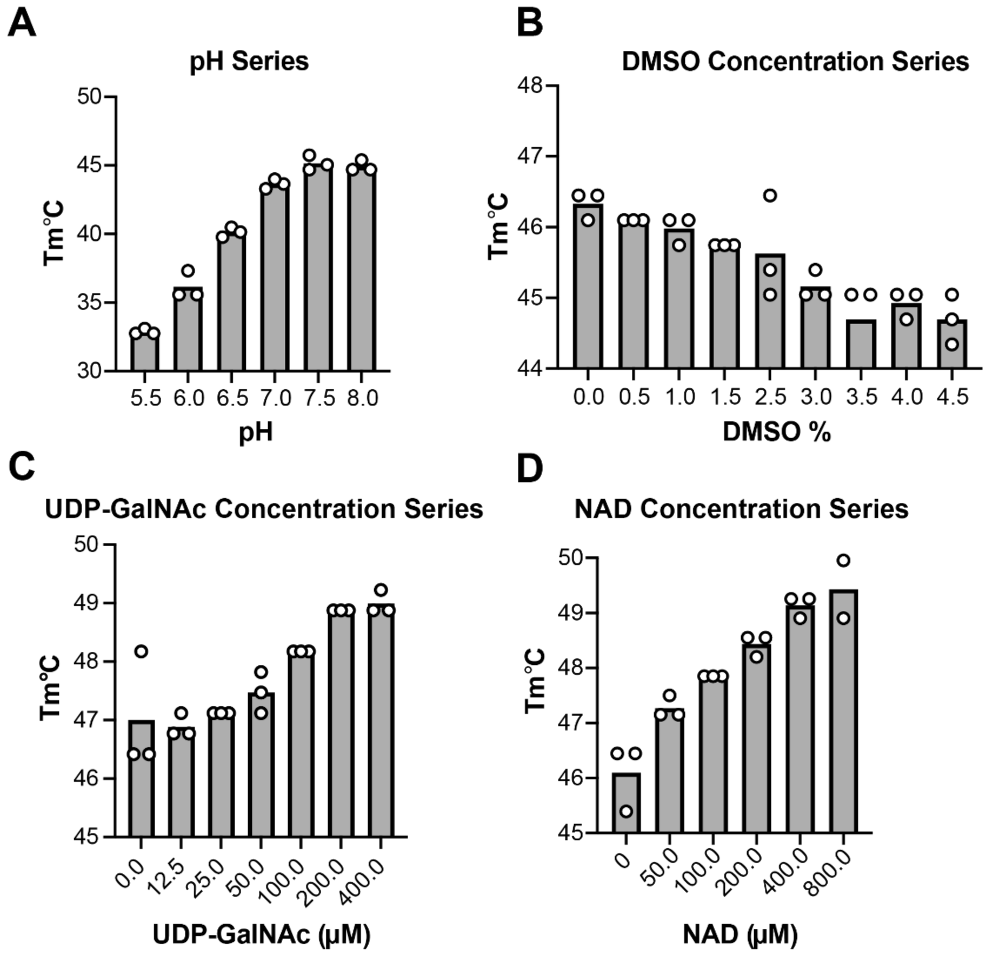

Fig. S2: Assessment of the DSF assay of recombinant GalE, with varying A) pH; B) DMSO concentration; C) UDP-GalNAc concentration; D) NAD concentration. Data are means of 2-3 technical replicates.

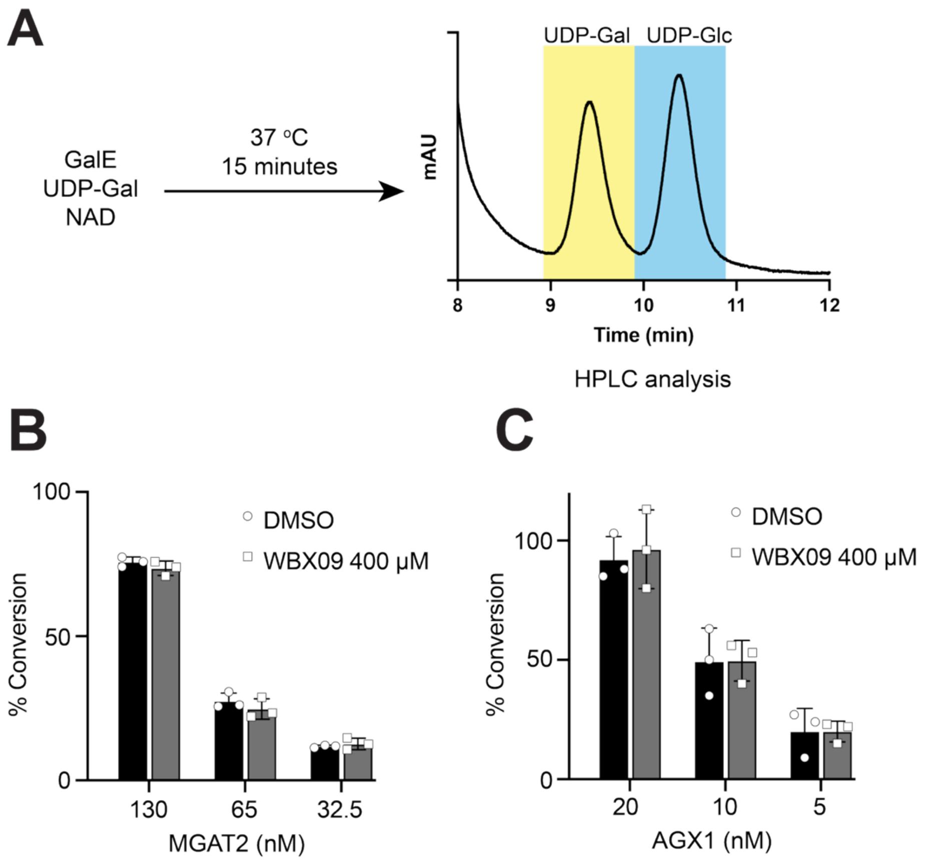

Fig. S3: A) Illustration of the epimerisation assay workflow. B and C) Effect on MGAT2 (B) and AGX1 (C) enzyme activity of WBX09 compared to DMSO only control at three enzyme concentrations. Data show individual data and means ± S. D. of three independent experiments.

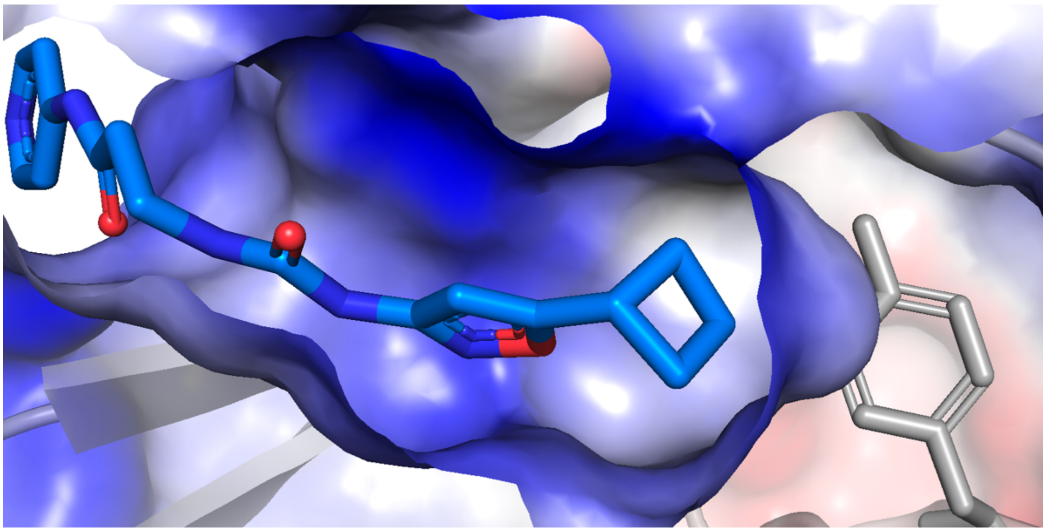

Fig. S4: Surface representation of the interaction between GalE and WBX09. Electrostatic surface is coloured (blue: positive, red: negative, grey: neutral/hydrophobic). WBX09 and Tyr230 are shown in stick representation. Structure displayed using Pymol v3.1.6.1 (Schrödinger).

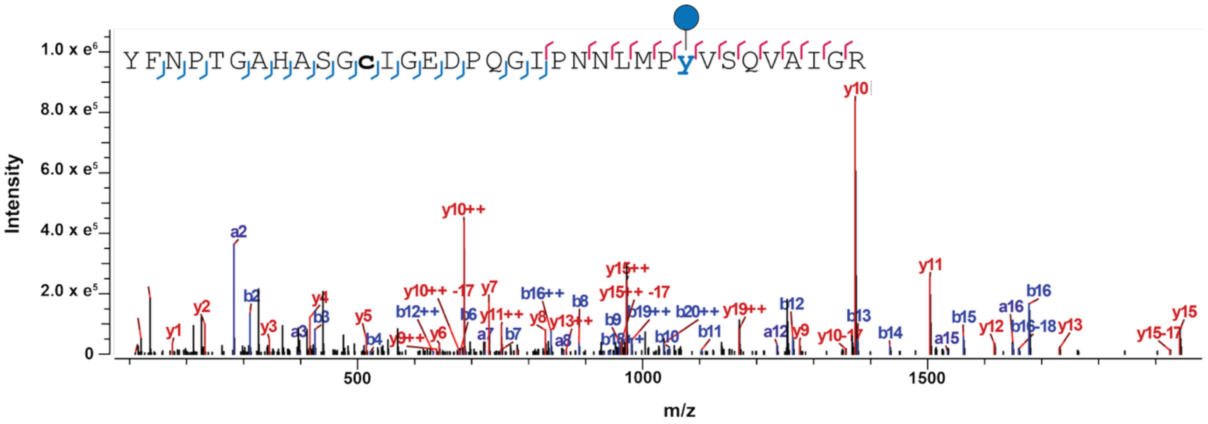

Fig. S5: Representative tryptic digest LCMS/MS spectra for a Tyr211-containing peptide modified by fragment WBC01. b ions are shown in blue and y ions in red. Lower case denotes modification. Blue sphere signifies covalent adduct. Spectra generated in Byonic (ProteinMetrics, v4.0.12). Data are from one experiment.

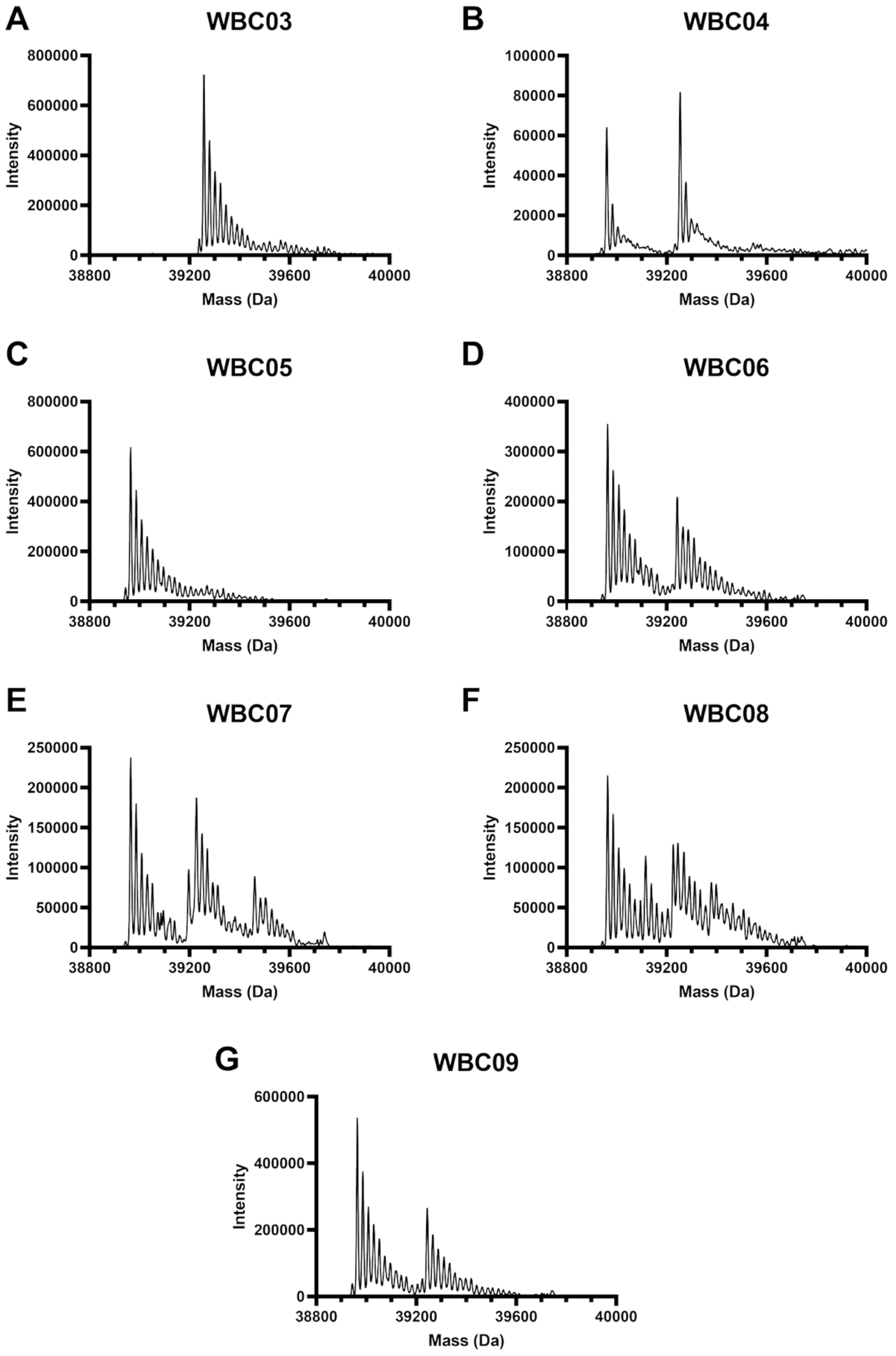

Fig. S6: Deconvoluted Intact-LCMS spectra for covalent compounds WBC03 (A) – WBC09 (G). 100 µM covalent compound, 0.5 µM GalE, 24-hour incubation. Data are from one experiment.

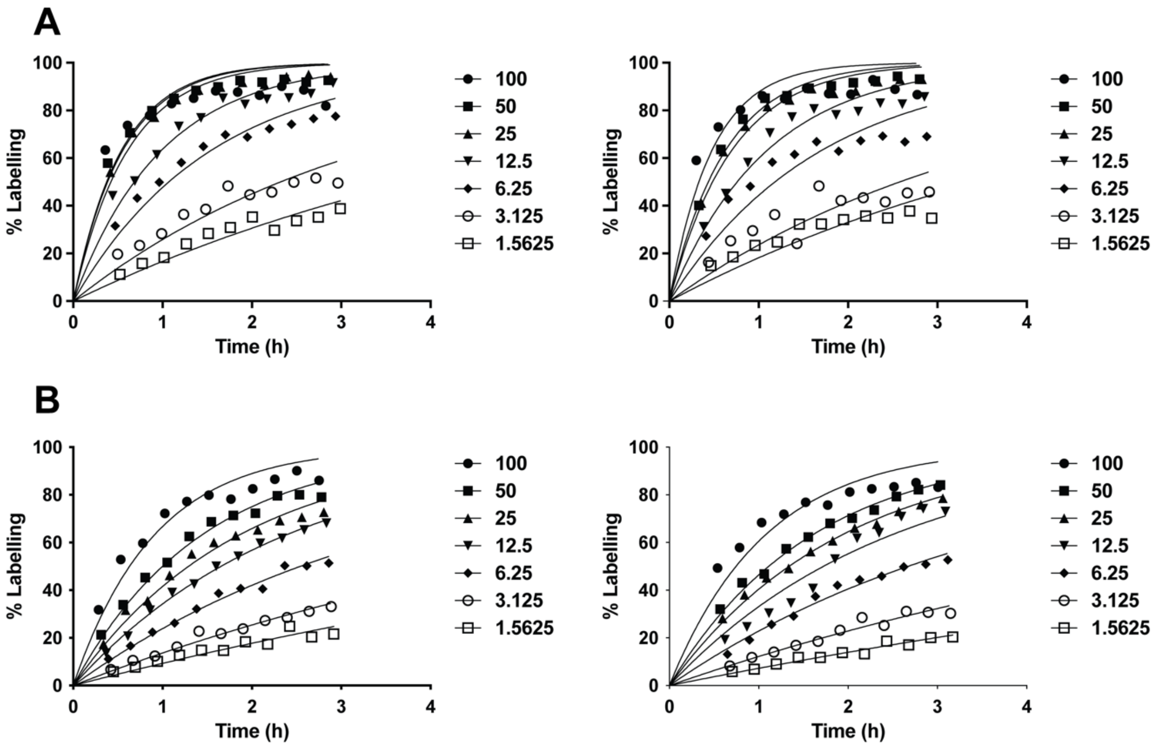

Fig. S7: Two independent replicates (left and right) of a GalE intact-LCMS time course experiment for compounds WBC10 (A) and WBC03 (B). Concentrations in legend are in µM.

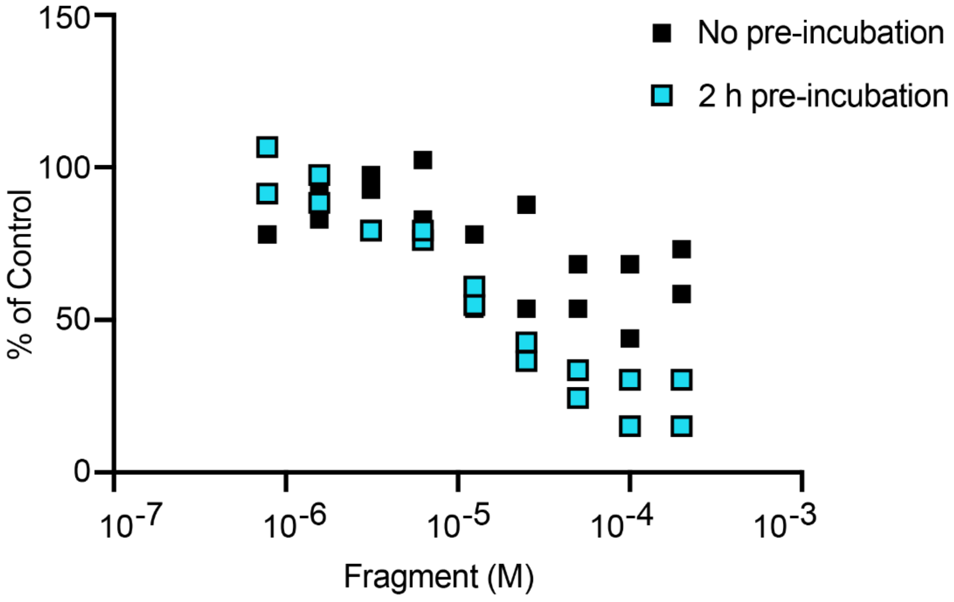

Fig. S8: Concentration-response curve for GalE with or without a 2-hour preincubation with >95% purity WBC10 at varying concentrations. Protein incubation performed as one experiment with biochemical assays performed in two technical replicates.

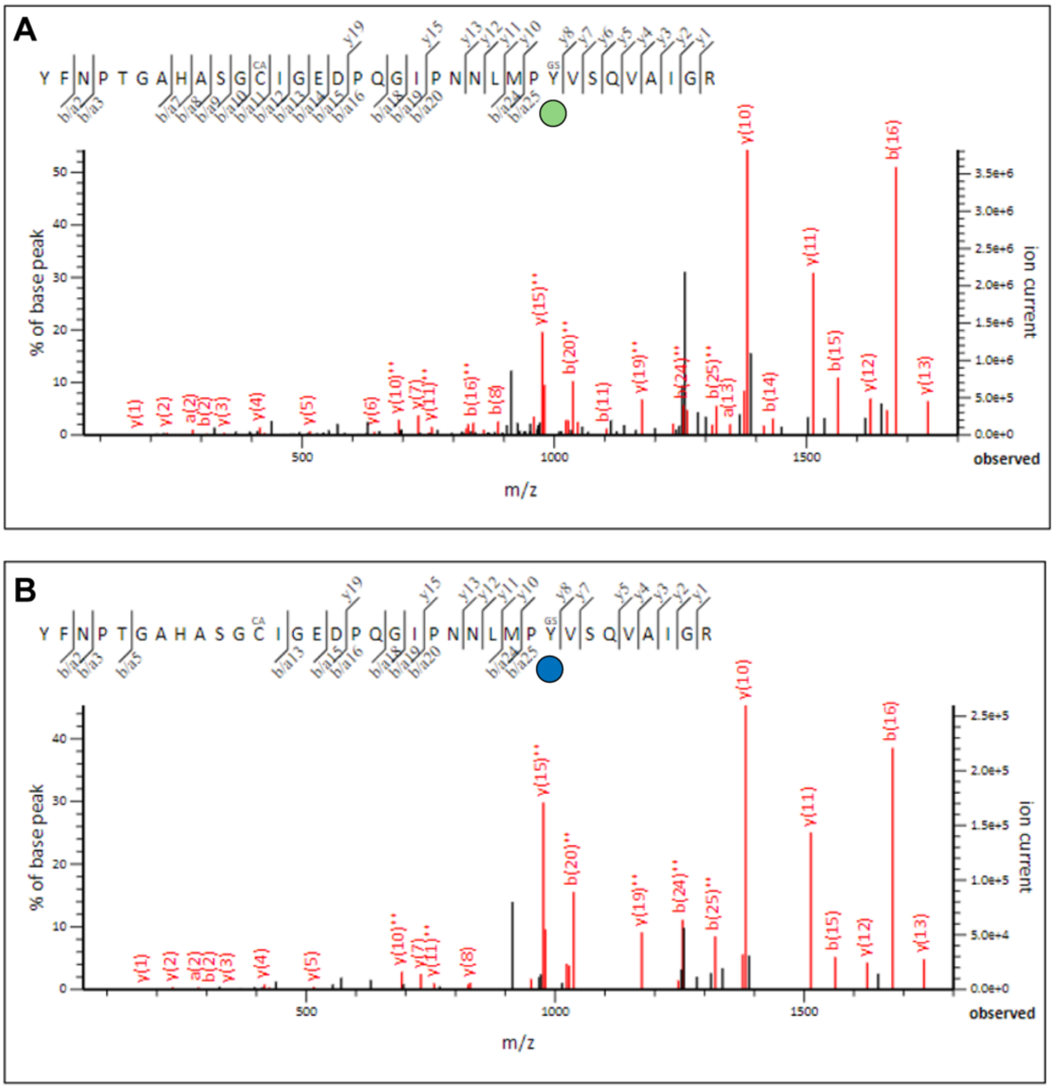

Fig. S9: Representative tryptic digest LCMS/MS spectra for a Tyr211 containing peptide modified by fragment WBC03 (A) and WBC10 (B). Modified Tyr211 represented by green (WBC03) and blue (WBC10) spheres. Data are from one experiment.

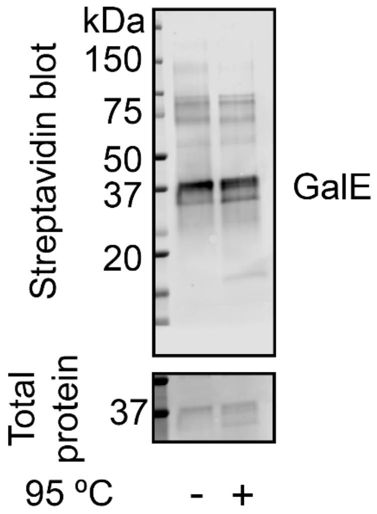

Fig. S10: Assessment of the stability of WBC11-GalE adduct. GalE was treated with WBC11 and subjected to SDS buffer at pH 6.8 for 5 min at 95 ºC. The adduct was subjected to CuAAC with biotin picolyl azide and streptavidin blot. Data are from one out of three independent replicates.

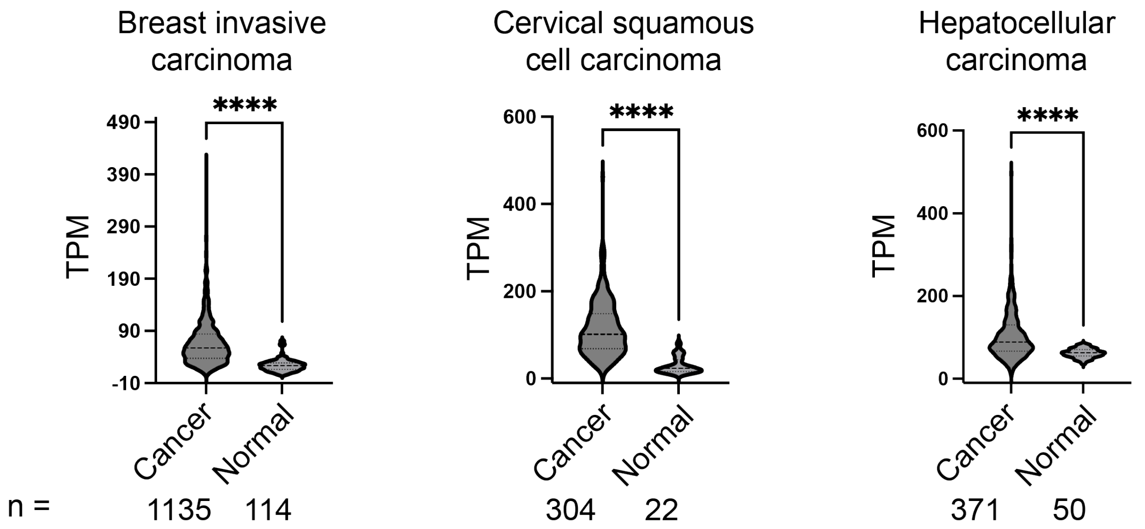

Fig. S11: Expression data of GalE in three cancer types according to OncoDB.org (accessed 13th Jan 2026). TPM = transcripts per million. Asterisks denote significance according to Welsh's *t*-test, **** *P* < 0.0001

Inhibitors and Screening Compounds

Fragments used for crystallographic screening were supplied by XChem at Diamond Light Source (DLS) and were part of the Enamine DSI-Poised library.

Covalent screening compounds were provided from an internal GSK library.

Compounds WBX01-14 (excluding WBX09) were purchased from Enamine and appear in their catalogue. WBX09 was custom synthesised by Enamine and their identity and purity confirmed by NMR in-house.

Covalent compounds WBC03-09 were also purchased from Enamine and appear in catalogue. WBC01 and WBC02 were screening compounds from internal GSK libraries. WBC10 and WBC11 were custom synthesised by Enamine, and the purity confirmed by NMR.

Methods

Protein Expression and Purification

Human GalE expression in Sf21 insect cells using the baculovirus expression system was described before.[1] For large-scale expression for crystallography, expression was established in BL21 (DE3) *E. coli* cells (Invitrogen). 1L of ZYM5052 autoinduction media was inoculated with one colony, incubated for 10h at 37 °C followed by 24h at 20 °C. Cells were harvested and resuspended in lysis buffer (50 mM HEPES, pH 7.5, 10 mM imidazole, 500 mM NaCl, 0.2% v/v Tween 20, 1 mM TCEP, 0.5 mM AEBSF, 15 µg/ml benzamidine, 10% glycerol and cOmplete EDTA-free protease inhibitor tablets (Roche, Basel, Switzerland)), lysed by French press, clarified, and stored at -80 °C. A HisTrap column (5 mL) was equilibrated in 5 column volumes (CV) of Buffer A (50 mM HEPES pH 7.5, 500 mM NaCl, 10 mM imidazole). GalE lysate was loaded into the column followed by a 20 CV wash step with Buffer A. The protein was eluted with 5 CV of buffer A supplemented with 250 mM imidazole. GalE was then concentrated to 5 mL and loaded onto a Superdex 200 26/60 column (Cytiva, Marlborough, USA)

equilibrated in Buffer B (20 mM HEPES pH 7.5, 100 mM NaCl, 0.5 mM TCEP). The fractions with active GalE are pooled together and cleaved with His-3C protease. The sample was then loaded again on a HisTrap column in buffer A. The cleaved product was recovered in the flowthrough, using a Vivaspin20 10,000 MWCO column (Cytiva) and loaded onto a Superdex 75 26/600 column (Cytiva) equilibrated in Buffer B. Purified GalE was then concentrated to 15 mg/mL and stored at -80 °C.

Crystallography

High-throughput crystallographic screening

Recombinant GalE protein was concentrated to 20 mg/mL in 25 mM HEPES pH 7.5, 500 mM NaCl, 2 mM NAD. The protein was crystallised in MRC 3 drop plates (Swisssci, Zug, Switzerland) by sitting drop vapour diffusion. Drops consisted of 100 nL GalE/NAD with 90 nL of 100 mM EPPS, pH 7.5, 136 mM KCl, 19.3 % w/v PEG 8K precipitant, 10 nL of seed stock in precipitant, equilibrating with 40 µL of precipitant. Drops were dispensed by NT8 (Formulatrix, Bedford, USA). In total, 6 plates with crystals were grown. Wells containing appropriate crystals were selected using a crystal ranking system and fragments in ethylene glycol dispensed into the selected wells using an Echo 550 (Labcyte, San Jose, USA) to a final concentration of either 10 (607 fragments) or 20 mM (250 fragments). Crystals were left to soak with fragments for ~3 hours at 4 °C. Crystals were fished using a Shifter (Oxford Lab Technologies, Oxford, UK) frozen in liquid nitrogen and stored in pucks for data collection.[2]

Data for all crystals was collected on Beamline IO4 (Diamond Light. Source) by unattended data collection at 100 K. Datasets consisted of 1800 images with an oscillation of 0.1 °, exposure time of 40 ms per image per crystal. Data sets were indexed, scaled and merged with xia2.[3] Data was processed using the XChem pipeline and the full method is described: https://www.diamond.ac.uk/Instruments/Mx/Fragment-Screening/The-XChem-Pipeline.[4],[5] However in summary, Xia2 auto-processed files were imported in the XChem explorer (XCE) pipeline where batch molecular replacement was first

performed on protein crystal only controls using DIMPLE and the “mixed homodimer” search model solved for GalE previously.[6] A ground-state model was then determined for GalE from these structures using the Pan-Dataset Density Analysis (PanDDA) method.[7] Ligands models and geometry restraints were prepared from the Enamine DSI-Poised library using AceDRG.[8] Batch molecular replacement using DIMPLE is then performed on the protein/fragment samples using the ground state structure. Hits are identified from unfilled density “blobs” using PanDDA and refinement performed with XCE.

WBX04, WBX09, X0213 co-crystallisation with GalE

GalE was crystallised at 20 °C using sitting-drop vapour diffusion. Sitting drops of 1 µL consisted of a 1:1:0.2 (V:V:V) mixture of protein, well solution and GalE seed stock. Well solutions were as follows 16% (V/V) PEG 3350, 0.2 M sodium malonate pH 7.0. Crystals appeared within a few hours and reached their maximum size after 2 to 3 days.

WBC10 co-crystallisation with GalE

GalE was covalently modified by incubating 10 µM of GalE in WBC buffer (50 mM HEPES-KOH pH 7.5, 50 mM NaCl, 2 mM NAD) with 100 µM covalent compound WBC10 (1% DMSO) overnight at room temperature. The sample was filtered and concentrated using a Amicon® Ultra Centrifugal Filter (Millipore, Burlington, USA) 10 kDa MWCO to 16.246 mg/mL.

GalE-WBC10 was crystallised at 20 °C using sitting-drop vapour diffusion. Sitting drops of 1 µL consisted of a 1:1:0.2 (V:V:V) mixture of protein, well solution and GalE seed stock. Well solutions were 22.6% PEG3350, 0.2 M $NaSO_4$, 0.1 M Bis-Tris pH 7.0. Crystals were cryoprotected in well solution supplemented with 30% (V/V) ethylene glycol.

Data collection

All crystals were cryoprotected in well solution supplemented with 30% (V/V) ethylene glycol and then soaked for 30 min in the cryoprotectant solution and 5 mM compounds. Crystals were then flash-frozen in liquid nitrogen and X-ray data were collected by unattended data collection at 100 K at beamlines I04 1 and I03 of the Diamond Light Source Synchrotron (Oxford, UK). Data collection and refinement statistics are summarised in section S10. Data sets were indexed, scaled and merged with xia2.[3] Molecular replacement used the atomic coordinates of human GalE from PDB 1EK5 in PHASER (McCoy et al., 2007).[9] Refinement used Phenix.[10] Model building used COOT with validation by PROCHECK.[11,12]

GalE Epimerisation Assay

In vitro epimerisation

For *in vitro* epimerisation, 20 µL reactions were performed in 25 mM HEPES, pH 7.5, 50 mM NaCl, 200 µM NAD with 100 µM UDP-Gal and 5 nM GalE. Reactions were run at 37 °C for 15 minutes. Quenching was carried out by heat inactivation at 95 °C for 1 minute. Samples were cooled to 4 °C and analysed by high performance liquid chromatography (HPLC) using an ArcPremier Waters instrument with a Dionex CarboPac PA1 IC (2mm x 150 mM) column (Waters, Wilmslow, UK). Injection volume was 5 µL. Eluent A was 1 M NaOAc/1 mM NaOH (Sigma-Aldrich, St. Louis, USA). Eluent B was 1 mM NaOH. The gradient was set to 60% A (0 min), 64% A (4 min), 68% A (8 min), 70% A (10 min), 72% A (12 min), 72% A (14 min), 76% A (16 min), 82%A (22 min), 82% A (27 min). The % conversion for each assay was determined by the relative difference in peak area between UDP-Gal and UDP-Glc peaks. Kinetics factors (K_M and k_{cat}) were determined by non-linear curve fitting using Graphpad Prism 9.0 GraphPad Software. Calculations used a modified version of the Michaelis-Menten equation (**Equation 1**).

$$\nu(GALE) = \frac{[GALE]_t \cdot k_{cat} \cdot [UDP - Gal]}{K_m + [UDP - Gal]}$$

Equation 1: Michaelis-Menten Equation as used in GraphPad.

Where ν is the initial rate in μM/s.
$K_m$is the Michaelis-Menten constant in μM.
$[GALE]_t$ is the concentration of GalE in μM in the assay.
$k_{cat}$is the turnover number in s^{-1}.
$[UDP - Gal]$ is the concentration of UDP-Gal at the start of the reaction in μM.

Non-Covalent Compound Concentration-Response Assay

Fragments were dispensed in a 2-fold dilution series from 1600 μM to 12.5 μM using an Echo 550 (Labcyte) into 384-well PCR plates (Applied Biosystems, Waltham, USA) from 100 mM DMSO fragment stocks. DMSO volume was adjusted to 1.6% (V/V) final concentration. Reactions were prepared in technical duplicates with triplicate DMSO only-controls and activity assessed by epimerisation assay.

Covalent GalE Inhibition Assay

A 2-fold dilution series of WBC10 (200 – 0.78 μM, 9 dilutions) was prepared in 25 nM GalE, 25 mM HEPES, pH 7.5, 50 mM NaCl, 200 μM NAD, 2% DMSO using 384-well PCR plates (Applied Biosystems) at room temperature. Assays were performed at the beginning of the incubation and 2 hours post WBC10 addition by making a 10-fold dilution of the incubation mixture in 25 mM HEPES, pH 7.5, 50 mM NaCl, 100 μM UDP-Gal and incubating at 37 °C for 10 minutes. Samples were heat inactivated at 95 °C for 1 min.

WBX09 MGAT2 and AGX1 Inhibition Assays

MGAT2 was recombinantly expressed before.[13] Reaction mixtures were prepared by addition of 130, 65 or 32.5 nM of MGAT2, 0.1 mM of procainamide-labelled acceptor substrate (A1-PROC, Ludger Ltd), 0.4 mM of UDP-GlcNAc and 0.4 mM of WBX09 or an equivalent volume of DMSO, in a total of 10 μL reaction buffer (50 mM Tris pH 6.8, with 5 mM $MnCl_2$, 0.1% BSA and 0.1% Triton-X100). Reactions were carried out at 37°C for 30 min and quenched with 10 μL acetonitrile. The reaction mixture was centrifuged at for 30 min at 14,000 *g* at 4°C to precipitate proteins. Of each sample, 9

µL of the supernatant was injected onto an Acquity H-Class PLUS QDa UPLC-MS (Waters) equipped with an ACQUITY UPLC® Glycan BEH Amide column (130 Å, 1.7 µm, 2.1 x 100 mm, Waters). Samples were run at flow rate of 0.35 mL/min using Buffer MGAT2_A (10 mM Ammonium formate at pH 4.5); Buffer MGAT2_B (10 mM Ammonium formate in ACN: water 90:10 (v/v)) with a gradient of 90-55% (V/V) Buffer MGAT2_B over 17 min. The turnover of the procainamide acceptor glycan into the product glycan was calculated by integration of the near-UV trace (302 nm absorption) of the acceptor substrate and the product glycan.

Recombinant AGX1 was expressed before.[14] Commercial *N*-Acetyl-D-glucosamine 1-phosphate disodium salt (GlcNAc-1-P, Sigma Aldrich) was used as a substrate to assess conversion to UDP-GlcNAc in the presence or absence of WBX09 by a UV absorbance-based assay. Reactions were carried out using 0.5 mM GlcNAc-1-P, 1 mM UTP (Sigma Aldrich), 0.4 mM WBX09 or an equivalent volume of DMSO and either 5 nM, 10 nM or 20 nM AGX1 in a total volume of 20 µL reaction buffer (5 mM $MgCl_2$, 25 mM Tris pH 8 and 1 mg/mL bovine serum albumin) at 37 °C for 90 min. Reactions were stopped by the addition of an equal volume of ice-cold acetonitrile, followed by centrifugation at 16200 *g*, 4 °C for 30 min. The supernatant of each reaction was analysed by injecting 9 µL into Acquity H-Class PLUS QDa UPLC-MS (Waters, Milford, USA) equipped with an ACQUITY UPLC® Glycan BEH Amide column (130 Å, 1.7 µm, 2.1 x 100 mm, Waters). Samples were run at a flow rate of 0.35 mL/min, column temperature at 50 °C and sample manager (sample tray) at 10 °C using Buffer AGX1_A (10 mM ammonium formate at pH 4.5); Buffer AGX1_B (10 mM ammonium formate in ACN: water 90:10 (V:V)) with a gradient of 90-55% (V/V) Buffer AGX1_B over 17 min. Product formation was monitored by absorption at 260 nm and further confirmed by mass detection in negative mode. Turnover (%) was determined by integration of the product's UV peaks and plotting it against a standard curve of 0-2.5 mM UDP-GlcNAc (Sigma- Aldrich) produced by serial dilution in final assay buffer.

Intact-LCMS Assay

Intact-LCMS was performed as described by Aatkar et al. with some modification, the following methods are adapted from this publication.[15] A 5 µL sample was injected

into an intact protein mass spectrometer with the following setup: an Agilent G230B time-of-flight (ToF) Accurate Mass Series spectrometer, an Agilent Bio-HPLC PLRP-S (1000 Å, 5 µM x 50 mm x 1 mm) reverse phase column, and an Agilent Dual AJS ESI ion source (Agilent Technologies, Cheshire, UK).

The flow rate and temperature were set to 0.5 mL/min and 70 °C, respectively. The solvent system used was as follows; solvent MS_A (water, 0.2% (V/V) formic acid), solvent MS_B (acetonitrile, 0.2% (V/V) formic acid). The applied gradient was 20% MS_B (0 min), 20% MS_B (0.6 min), 50% MS_B (0.61 min), 100% MS_B (1 min), 100% MS_B (1.2 min), 20% MS_B (1.21 min). For data acquisition, positive ion scanning was performed between 600 – 3200 Da with a scan rate of 1.2 s. The obtained spectra were analysed and deconvoluted with the MassHunter BioConfirm software (Agilent Technologies, Cheshire, UK). The expected mass range used was 30000 – 50000 Da. The spectra were then exported and analysed using software developed internally at GSK to determine relative % labelling. The equation used to determine labelling is shown in **Equation 2**.

$$\%\,Modification = \frac{Intensity\ of modified\ protein}{(Intensity\ of\ protein\ only + Intensity\ of\ modified\ protein)} x100$$

Equation 2: Calculation used to determine% protein labelling from intact-LCMS assays.

Covalent Fragment Intact-LCMS Screening:

Covalent fragments (100 µM fragment, 1% DMSO final) were dispensed by Echo 550 into a Greiner 384 well plate (Sigma Aldrich) followed by the addition of GalE (0.5 µM, 25 mM HEPES, pH 7.5, 50 mM NaCl) and incubation for 24 hours at 4 °C. DMSO only controls were included. Samples were analysed by intact-LCMS as described above and the relative % protein modification determined. Full data output and all deconvoluted LCMS spectra are available on request.

Covalent Fragment Kinetics Assay:

Fragments were first dispensed by Echo 550 into a Greiner 384 well plate to provide a twofold dilution series from 100 µM to 1.56 µM in 120 µL of GalE (0.5 µM, 25 mM HEPES, pH 7.5, 50 mM NaCl,) at room temperature. Each sample was assessed consecutively by intact-LCMS.

Isothermal Titration Calorimetry Assay

ITC characterisation was performed using a MicroCal PEAQ ITC system (Malvern Panalytical, Great Malvern, UK) at 25 °C. 300 µL recombinant human GalE at 25 µM in 25 mM HEPES, pH 7.5, 50 mM NaCl, 4 % DMSO buffer, was loaded into the sample cell. Ligands were prepared at 4 mM or 250 µM in buffer matching the protein. Ligands were titrated into the cell with 1x0.4 µL injection followed by 19x2 µL injections, 4 s injection durations with 150 s spacing. Heat release per titration was measured and integrated using the MicroCal PEAQ ITC software. All kinetic parameters such as K_D, Enthalpy (ΔH), Gibbs free energy (ΔG), and stoichiometry (N) were determined using a single site occupancy model.

Differential Scanning Fluorimetry Thermal Shift Assay

Premixed protein-dye was first prepared at 4 °C as follows: 2 µM GalE, 5X SYPRO orange dye (ThermoFisher, Waltham, USA), 2 mM NAD, 25 mM HEPES, pH 7.5, 50 mM NaCl. Fragments were dispensed with an Echo 550 into 384-well PCR plates (Applied Biosystems). 20 µL of protein-dye mix was dispensed into each well using a VIAFILL dispenser (Integra, Princeton, USA). Plates were then spun at 1000 rpm for 1 minute and incubated at 4 °C for 10 minutes. Melt curves were acquired using a QuantStudio 12K Flex Real-Time PCR System (ThermoFisher). A temperature gradient was run from 25-95 °C (3 °C/min). Acquired melt curves were analysed using the Protein Thermal Shift™ Software (Applied Biosystems). For DSF optimization, variations in pH, DMSO concentration, UDP-GalNAc and NAD concentration were performed.

***In vitro* DMPK**

Hepatocyte stability assay and MDCK permeability assay of WBX09 were performed by Pharmidex (London, UK).

Nuclear Magnetic Resonance

^{1}H and ^{13}C NMR spectra were measured with Bruker Avance-400 or 600 NMR spectrometers at 298 K at the Francis Crick Institute. Chemical shifts (σ) are reported in parts per million (ppm) relative to the respective residual solvent peaks ($CDCl_3$-TMS: σ 0.00 in ^{1}H and 77.16 in ^{13}C NMR; DMSO-d6: σ 2.50 in ^{1}H and 39.51 in ^{13}C NMR). The following abbreviations are used to indicate peak multiplicities: s singlet; d doublet; dd doublet of doublets; m multiplet. Coupling constants (J) are reported in Hertz (Hz).

Virtual Docking

ICM-Pro (Molsoft, San Diego, USA) was used for virtual docking. First, ICM receptor maps for a specified binding site were made from X-ray structures (either 1EK5 or 9HJN) employing five types of interaction potentials: 1) Van der Waals interactions for hydrogen probes, 2) Van der Waals interactions for heavy atoms, 3) hydrophobic terms, 4) lone-pair-dependent H-bonding potentials, and 5) electrostatic terms.[16] The force field (FF) used for this task is the Empirical Conformational Energy Program for Peptides (ECEPP).[17] Ligand conformational docking within the receptor maps uses the biased probability Monte Carlo method.[18] The number of moves is determined by an algorithm influenced by the chosen "Thoroughness" value (set to 3 for all screening iterations). The ligands are modelled based on the Merck Molecular Force Field (MMFF).[19] The ICM score was used to rank docked ligands.

Docking was performed as follows:

- Import of the X0213 GalE crystal structure.
- Conversion of the PDB file to an ICM object using the ICM method.

- Options: Optimisation of hydrogens, keep tight waters (those that make 3 or more H-bonds), optimise His, Pro, Asn, Gln, and Cys side chains.
- Options: Optimisation of formal and partial charges, protonation states (at pH 7.5) and hydrogen placement.
- Identification of the binding pocket and building of receptor maps around X0213 using the ICM virtual screening function.
- Docking of libraries from SDFiles using the ICM virtual ligand screening function, selecting a thoroughness score of 3.
- All other docking parameters kept as default, and no additional triaging was done.

WBC01 and WBC02 Covalent Fragment Site Identification Mass Spectrometry

Recombinant human GalE (10 µM GalE, 25 mM HEPES, 50 mM NaCl) was first incubated with either WBC01 or WBC02 (25, 50, or 100 µM) or matched 1% (V/V) DMSO control for 24 hours at room temperature. Samples were separated by SDS-PAGE gel, stained with colloidal Coomassie InstantBlue (Abcam, Cambridge, UK), bands corresponding to GalE were excised, reduced in 10 mM DTT (in water) for 20 minutes at 55 °C, alkylated with 55 mM iodoacetamide (IAA) in the same buffer at room temperature for 20 minutes, and digested overnight using 100 ng trypsin in 10 mM ammonium bicarbonate at 37 °C (Promega, Wisconsin, USA) before being acidified with to 0.1% Trifluoracetic acid (TFA).

On an Ultimate 3000 nanoRSLC HPLC (Thermo Scientific, Massachusetts, USA), 1-10 µL of protein sample was loaded on a 20 mm x 75 µm Pepmap C18 trap column (Thermo Scientific) prior to elution via a 50 cm x 75 µm EasySpray C18 column into a Lumos Tribrid Orbitrap mass spectrometer (Thermo Scientific). A 45 minute gradient of 6% - 40% B was used followed by washing and re-equilibration (A= 2% ACN, 5% DMSO, 0.1% formic acid; B= 80% ACN, 5% DMSO, 0.1% formic acid). The Orbitrap was operated in “Data Dependent Acquisition” mode followed by MS/MS in “TopS” mode, with Orbitrap accumulation at R=30K of higher-energy C-trap dissociation (HCD) fragmented parent ions.

Data evaluation was performed with Byonic™ (Protein Metrics, Cupertino, USA, version 4.0.12). For covalent compound residue modification search parameters included semi-specific cleavage specificity at the C-terminal site of R and K, with two missed cleavages allowed. Mass tolerance was set at 10 ppm for MS1s, 20 ppm for HCD MS2s, and 0.2 Da for ETD MS2s. Carbamidomethyl cysteine was set as a fixed modification. Variable modifications included methionine oxidation (common 1), asparagine deamidation (common 1), and custom modifications for the addition of WBC01 and WBC02 on either tyrosines or lysines. A maximum of two variable modifications were allowed per peptide. For each sample, variable modifications were searched against a focused FASTA file that exclusively contains protein sequences found in that sample.

WBC03 and WBC10 Covalent Fragment Site Identification Mass Spectrometry

The covalent compounds (WBC03 and WBC10) were plated into a Greiner 384 PP F-bottom plate from a 10 mM source in DMSO. Purified GalE diluted from original stock into a buffer made up of HEPES (pH 7.5, 25 mM) and NaCl (50 mM) in distilled MS-grade water (50 µL per well) was subsequently added across the plate. Final concentrations of protein and covalent compound were used as indicated. The plate was sealed, centrifuged (1 min, 1000 rpm), incubated at 20 °C for 6 h, and 15 µL aliquots were subsequently removed and analyzed by intact protein LC-MS to confirm modification. The remaining samples (1 µg) were separated by SDS-PAGE to remove excess unbound compound. Gels were stained with colloidal Coomassie InstantBlue and bands corresponding to GALE were excised, reduced with 10 mM TCEP (65 °C, 30 mins), and alkylated with 10 mM iodoacetamide (room temperature, 30 mins, dark). Samples were digested with trypsin/LysC mix (Promega, Wisconsin, USA) 1:10 E:S (37 °C, 16 h) in 25 mM ammonium bicarbonate. Supernatants were concentrated in a SpeedVac centrifuge and acidified (0.1% formic acid, 0.05% trifluoroacetic acid) prior to injection into the LCMS/MS system.

Digested samples were injected on an Easy-nLC 1000 UHPLC system (Thermo Scientific). The nanoLC was interfaced to a Q-Exactive Hybrid Quadrupole-Orbitrap Mass Spectrometer (Thermo Scientific). Tryptic peptides were loaded on a 2 cm × 75 µm Acclaim PepMap 100 C18 trapping column (Thermo Scientific) and separated on

a 25 cm × 75 µm, 2 µm particles, PepMap C18, 2 µm particle column (Thermo Scientific) using a 50 min gradient of 2–38% acetonitrile, 0.2% formic acid and a flow of 300 nL/min. LC-MS/MS based peptide sequencing was performed by data dependent analysis (DDA). Full MS 400–1600 Da at 70 000 resolution, MS AGC target 1e6, MS Maximum IT 200 ms, followed by MS/MS top 10 HCD fragmentation, stepped normalized CE 23, 27 and 30 V, Isolation window 1.5 m/z, fixed first mass 145 m/z, 17,500 resolution, MS/MS AGC target 5e4 and MS/MS Maximum IT 200 ms.

Uninterpreted tandem MS spectra were searched for peptide matches against the sequence for GALE using the Mascot (Version 2.6.0) software with a 5 ppm mass tolerance for peptide precursors and 20 mDa mass tolerance for fragment ions. Raw files were searched using trypsin as the enzyme with up to 2 missed cleavages and the variable modifications carbamidomethylation on cysteine and oxidation on methionine were allowed. Masses corresponding to [WBC03 or WBC10 − HF] (293.30 Da and 333.36 Da, respectively) were allowed as variable modification(s) on cysteine, histidine, lysine, tyrosine, serine, and threonine as well as the protein N-terminus. MS/MS spectra were manually validated and annotated.

WBC11 Alkyne protein labelling assay

Recombinant samples of either GalE or AGX1 (0.5 µg enzyme in 25 mM HEPES pH 7.5, 50 mM NaCl, 200 µM NAD) were incubated with either 10 µM WBC11 alkyne, 100 µM WBX09, or 10 µM WBC11 alkyne and 100 µM WBX09 in DMSO (1% DMSO in final conditions) for 1 hour at room temperature in 27 µL volume. Samples then underwent copper-catalyzed azide-alkyne cycloaddition (CuAAC) with 100 µM biotin picolyl azide (Sigma-Aldrich), 600 µM copper(II) sulfate, 1.2 mM 2-(4-((Bis((1-(*tert*-butyl)-1*H*-1,2,3-triazol-4-yl)methyl)amino)methyl)-1*H*-1,2,3-triazol-1-yl)acetic acid (BTTAA), 5 mM sodium ascorbate, and 5 mM amminoguanidine hydrochloride for 30 minutes at room temperature. Subsequently, a 4X sample buffer (333 mM Tris-HCl, pH 6.5, 26.7% glycerol, 3.33% SDS in de-ionised water) was added to a final 1X concentration. Stability of the covalent bond was assessed by incubating the GalE-WBC11 conjugate for 5 minutes at 95 ºC. Samples were run on an 4%-20% SDS PAGE gel (Criterion^{TM} TGX^{TM} Precast Midi Protein Gel, Bio-Rad) for 70 mins at 160V. Western blot transfer to 0.2 µm nitrocellulose membrane (Bio-Rad) was done with the

Transblot-Blot Turbo Transfer System (Bio-Rad) at the HIGH MW protocol (10 mins, 2.5 A, up to 25 V). For total protein staining, membrane was incubated with Revert 700 Total Protein stain (LI-COR) for 5 mins. Membrane was blocked for 1 hour with Intercept (TBS) Blocking Buffer (LI-COR) and subsequently stained with IRDye 800 CW Streptavidin (LI-COR) (1:5000 in blocking buffer, 0.05% SDS and 0.2% Tween20). All membrane scans were done with Odyssey CLx Imager (LI-COR) and images were analysed on Image Studio (version 6.0, LI-COR).

Densitometry analysis was done in Image Studio (version 6.0). Area around one band was defined via a manually drawn box, after which the same box was copied and used for all other bands. Background noise was set to be the median of all sides around each box, with border width set to 3. For normalisation, the signal of each total protein band was taken (signal from 700 nm channel). Each total protein signal value was then divided by the highest total protein signal value of that replicate, giving the normalisation factor for each band. The streptavidin signal for each band (800 nm channel) was then divided by its respective normalisation factor to obtain the final normalised densitometry score.

Crystallography Statistics

Table SI. 2: Crystallographic data and refinement statistics.

	X0213 (9HJN)	WX04 (9HI0)	WX09 (9HI1)	WBC10 (9HI2)
Resolution range (/Å)	43.74 - 1.65 (1.68-1.65)	68.18 - 1.37 (1.39 - 1.37)	57.69 – 0.95 (0.97 – 0.95)	57.07 – 1.37 (1.39 – 1.37)
Space group	P 2_1 2_1 2_1	P$2_12_12_1$	P$2_12_12_1$	C222_1
Unit cell (/Å) a, b, c	46.14, 08.33, 137.45	46.27 110.70 136.27	46.15 110.20 135.41	50.04 114.14 130.38
(/^{0}) α, β, γ	90, 90, 90	90 90 90	90 90 90	90 90 90
Total reflections	1171261 (59151)	10 549 630 (341 238)	17 547 146 (897 388)	297 983 (14 395)
Unique reflections	160 447 (7930)	143 992 (6 623)	415 408 (22 430)	77 616 (4 002)
Multiplicity	7.3(7.4)	73.3 (51.5)	40.0 (39.5)	3.8 (3.6)
Completeness (%)	99.4 (99.3)	97.4 (90.9)	99.4 (93.0)	99.0 (99.8)
Mean I/sigma(I)	6.1 (0.7)	12.5 (0.5)	76.5 (0.3)	7.9 (0.80)
Wilson B-factor	19.3	15.05	11.17	
R-merge	0.44 (8.29)	0.84 (5.45)	0.13 (14.27)	0.06 (1.50)
R-meas	(8.90)	0.84 (5.51)	0.135 (14.45)	0.08 (1.91)
R-pim	(3.22)	0.09 (0.76)	0.02 (2.26)	0.05 (0.98)
$CC_{1/2}$	0.987 (0.071)	0.96 (0.41)	1.0 (0.33)	1.0 (0.30)
Reflections used in refinement	83318	143 314 (4 177)	408 041 (9 840)	77 608 (2 839)
Reflections used for R-free	4221	7 044 (205)	7 068 (208)	3 945 (151)
R_{work}	0.219	0.15 (0.32)	0.17 (0.38)	0.14 (0.28)
R_{free}	0.190	0.19 (0.31)	0.17 (0.38)	0.17 (0.32)
Number of non-hydrogen atoms	5,830	6 052	6 341	3 060
macromolecules	5,281	5 369	5 576	2 672
ligands	112	159	184	67
solvent	437	524	581	321
Protein residues	686	689	687	344
RMS(bonds)	0.008	0.013	0.230	0.139
RMS(angles)	1.647	1.80	2.27	1.33
Ramachandran favoured (%)	98.0	97.52	97.66	97.65
Ramachandran allowed (%)	2.0	2.48	2.20	2.35
Ramachandran outliers (%)	0.8	0.00	0.15	0.00
Clash score	3.3	1.10	3.65	1.85
Average B-factor		23.18	15.02	21.54
macromolecules	23.0	22.04	13.98	20.14
ligands		23.96	17.89	18.95
solvent	29.9	34.66	24.08	33.69

Nuclear Magnetic Resonance spectra

WBX09

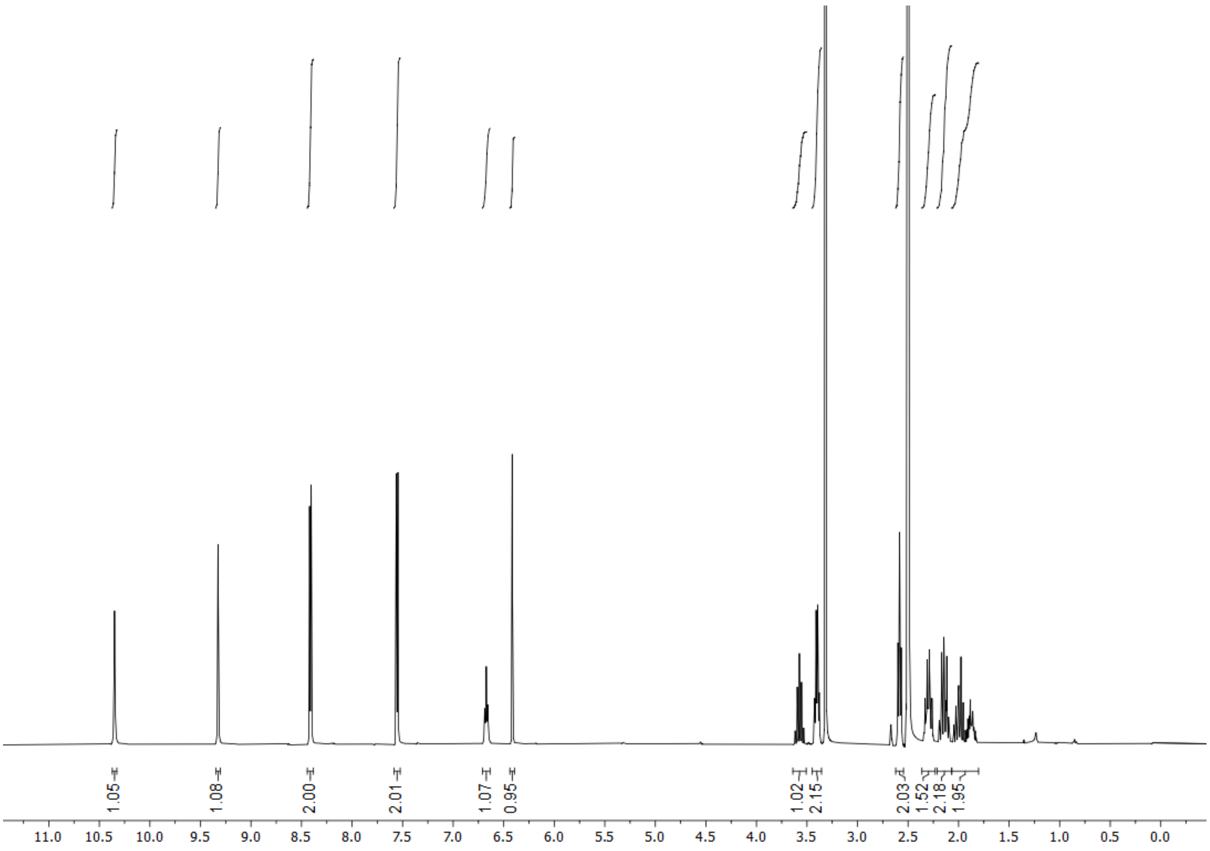

^{1}H NMR (400 MHz, DMSO-d6) δ = 10.35 (s, 1H), 9.33 (s, 1H), 8.44 – 8.39 (m, 2H), 7.59 – 7.52 (m, 2H), 6.67 (t, *J*=5.9, 1H), 6.42 (d, *J*=0.8, 1H), 3.63 – 3.52 (m, 1H), 3.40 (q, *J*=6.2, 2H), 2.58 (t, *J*=6.3, 2H), 2.35 – 2.25 (m, 2H), 2.20 – 2.08 (m, 2H), 2.05 – 1.94 (m, 1H), 1.94 – 1.82 (m, 1H).

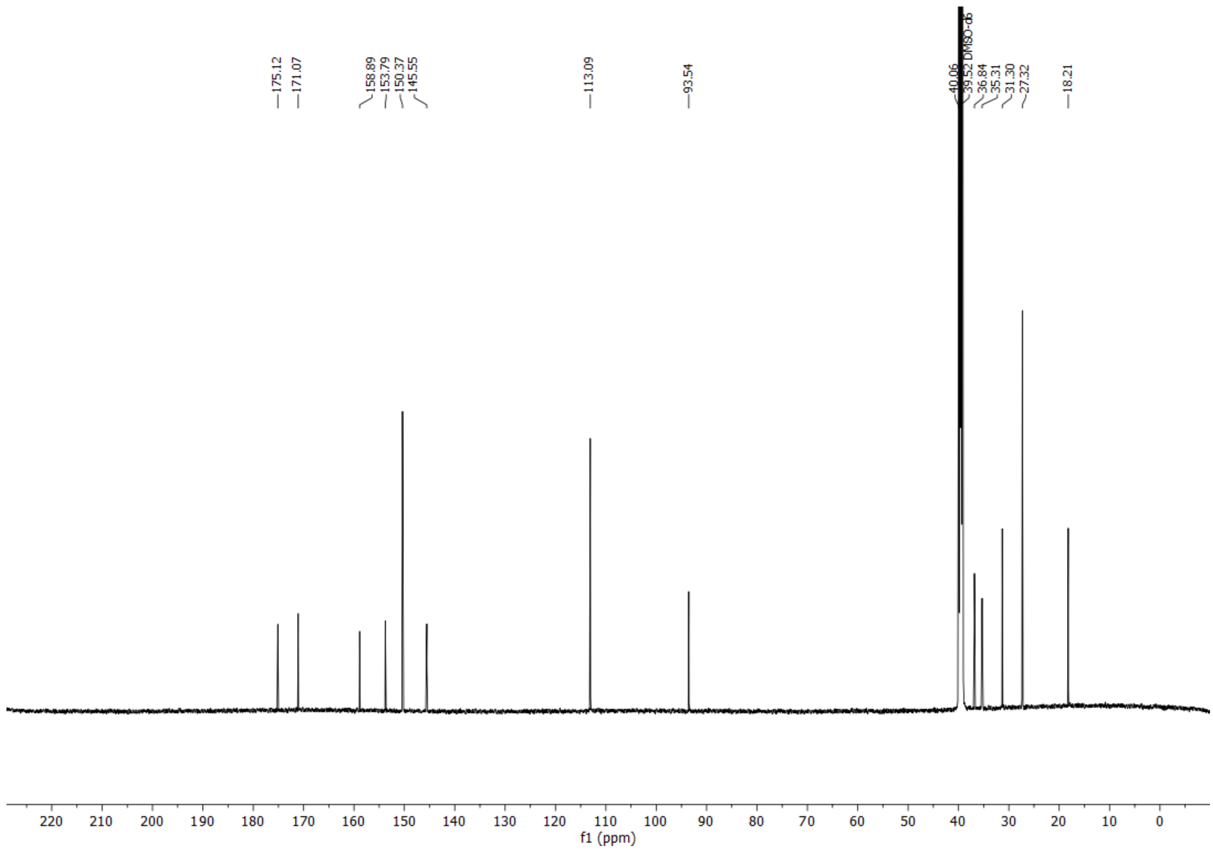

^{13}C NMR (150 MHz, DMSO-d6) δ = 175.6, 171.5, 159.4, 154.3, 150.8, 146.0, 113.6, 94.0, 40.5, 37.3, 35.8, 31.8, 27.8, 18.7.

WBC10

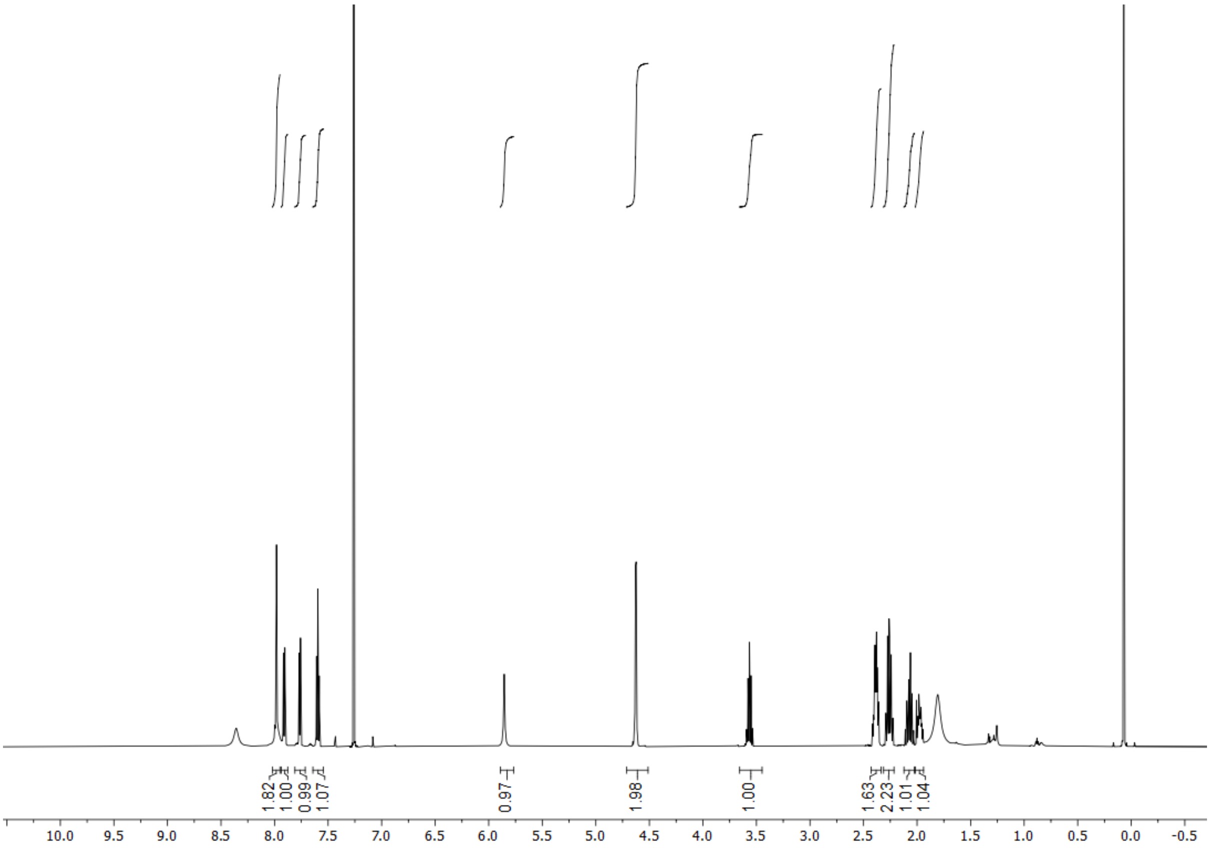

^{1}H NMR (600 MHz, $CDCl_3$ with TMS) δ = 8.01 – 7.94 (m, 1H), 7.91 (dd, *J*=7.9, 1.6, 1H), 7.76 (d, *J*=7.7, 1H), 7.60 (t, *J*=7.8, 1H), 5.86 (s, 1H), 4.70 – 4.54 (m, 2H), 3.56 (p, *J*=8.5, 1H), 2.44 – 2.32 (m, 2H), 2.30 – 2.20 (m, 2H), 2.07 (dp, *J*=11.3, 8.7, 1H), 2.02 – 1.94 (m, 1H).

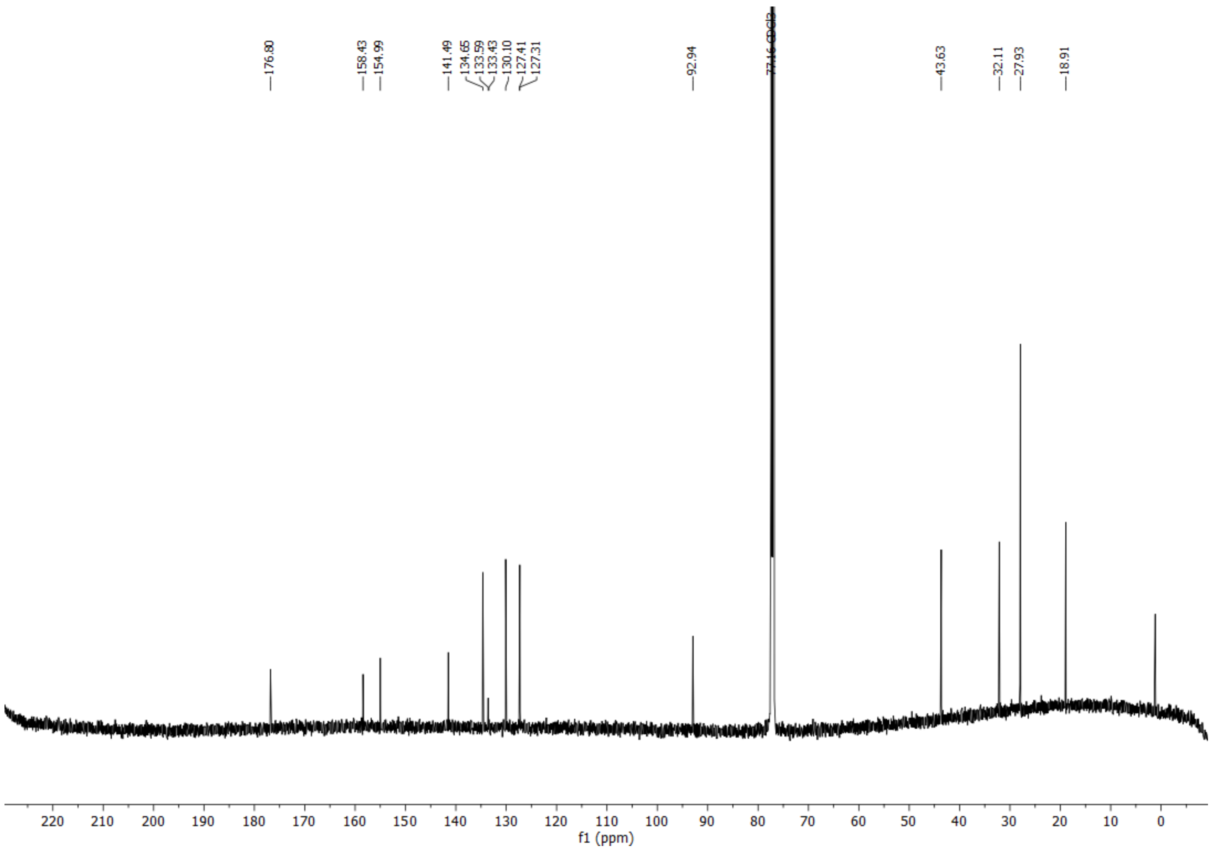

^{13}C NMR (150 MHz, $CDCl_3$ with TMS) δ = 176.8, 158.4, 155.0, 141.5, 134.7, 133.6, 133.4, 130.1, 127.4 (d, *J*=15.5), 92.9, 43.6, 32.1, 27.9, 18.9.

WBC11

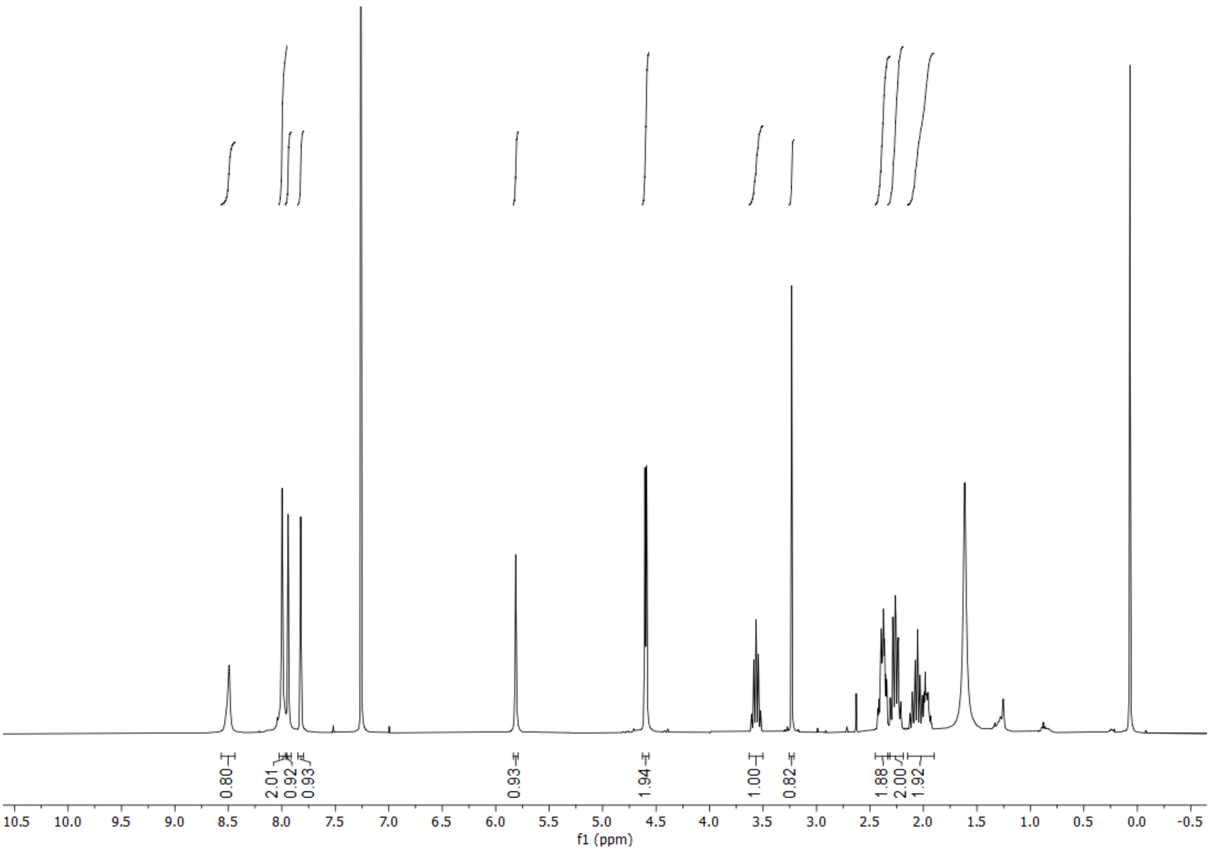

^{1}H NMR (400 MHz, $CDCl_3$ with TMS) δ = 8.43 (s, 1H), 7.93 (d, *J*=1.69, 1H), 7.88 (d, *J*=1.74, 1H), 7.76 (d, *J*=1.68, 1H), 5.75 (s, 1H), 4.53 (d, *J*=5.96, 2H), 3.50 (p, *J*=8.51, 1H), 3.16 (s, 1H), 2.32 (m, *J*=14.35, 10.61, 8.36, 4.30, 1.77, 2H), 2.26 – 2.12 (m, 2H), 2.08 – 1.83 (m, 2H).

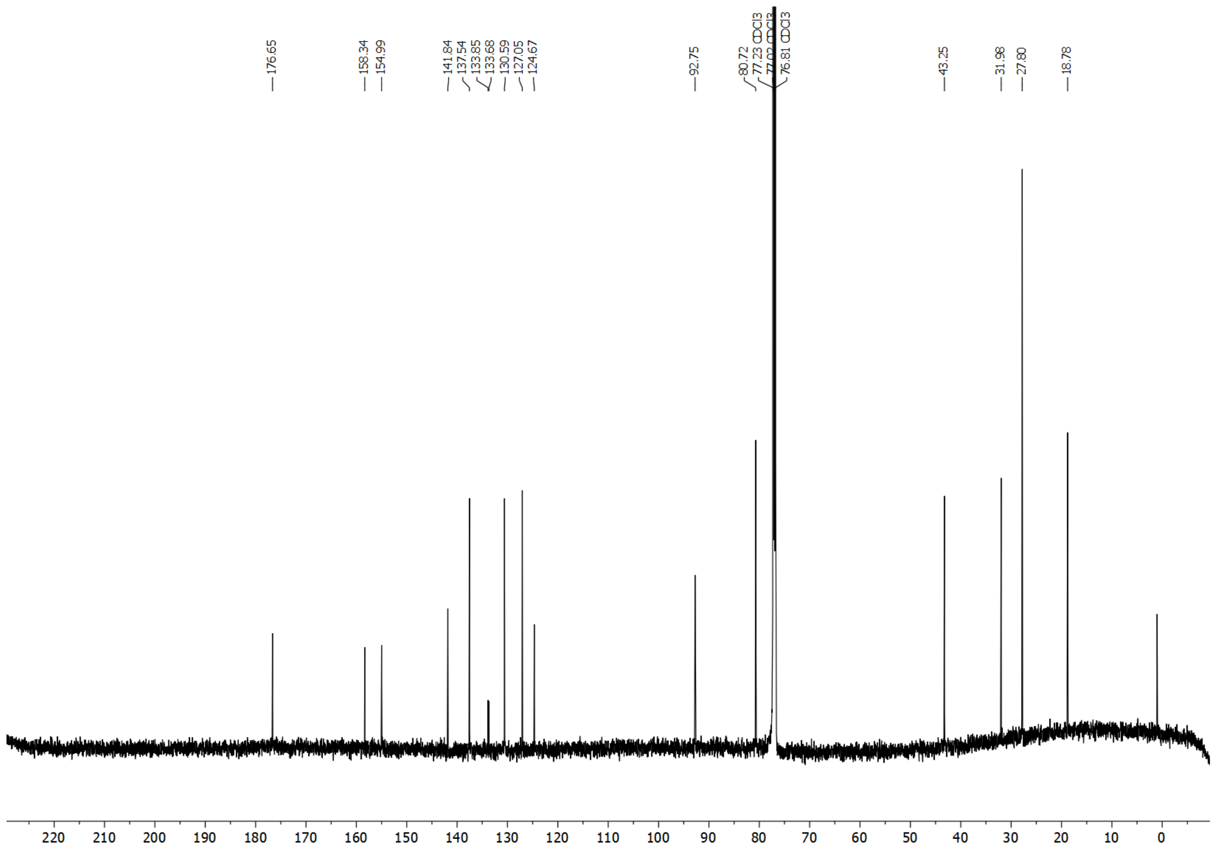

^{13}C NMR (150 MHz, $CDCl_3$ with TMS) δ = 176.6, 158.3, 155.0, 141.8, 137.5, 133.8, 133.7, 130.6, 127.1, 124.7, 92.8, 80.7, 43.2, 32.0, 27.8, 18.8.

References

1. M. F. Debets, O. Y. Tastan, S. P. Wisnovsky et al., Metabolic precision labeling enables selective probing of O-linked N-acetylgalactosamine glycosylation, *Proc. Natl. Acad. Sci. USA* **2020**, *117*, 25293-25301.
2. N. D. Wright, P. Collins, L. Koekemoer et al., The low-cost Shifter microscope stage transforms the speed and robustness of protein crystal harvesting, *Acta Crystallogr. D Struct. Biol.* **2021**, *77*, 62–74.
3. G. Winter, C. M. C. Lobley, S. M. Prince, Decision making in xia2, *Acta Crystallogr. D Biol. Crystallogr.* **2013**, *69*, 1260–1273.
4. The XChem Pipeline – Diamond Light Source, https://www.diamond.ac.uk/Instruments/Mx/Fragment-Screening/The-XChem-Pipeline.html (accessed November 10, 2023).
5. T. Krojer, R. Talon, N. Pearce et al., The XChemExplorer graphical workflow tool for routine or large-scale protein-ligand structure determination, *Acta Crystallogr. D Struct. Biol.* **2017**, *73*, 267–278.
6. M. Wojdyr, R. Keegan, G. Winter, A. Ashton, DIMPLE-a pipeline for the rapid generation of difference maps from protein crystals with putatively bound ligands, *Acta Crystallogr. A* **2013**, *69*, s299–s299.
7. N. M. Pearce, A. R. Bradley, T. Krojer, B. D. Marsden, C. M. Deane, F. von Delft, Partial-occupancy binders identified by the Pan-Dataset Density Analysis method offer new chemical opportunities and reveal cryptic binding sites, *Struct. Dyn.* **2017**, *4*, 032104.
8. F. Long, R. A. Nicholls, P. Emsley, S. Gražulis, A. Merkys, A. Vaitkus, G. N. Murshudov, AceDRG: a stereochemical description generator for ligands, *Acta Crystallogr. D Struct. Biol.* **2017**, *73*, 112–122.
9. A. J. McCoy, R. W. Grosse-Kunstleve, P. D. Adams, M. D. Winn, L. C. Storoni, R. J. Read, Phaser crystallographic software, *J. Appl. Crystallogr.* **2007**, *40*, 658-674.
10. P. D. Adams, P. V. Afonine, G. Bunkóczi, V. B. Chen, I. W. Davis, N. Echols, J. J. Headd, L. W. Hung, G. J. Kapral, R. W. Grosse-Kunstleve, A. J. McCoy, N. W. Moriarty, R. Oeffner, R. J. Read, D. C. Richardson, J. S. Richardson, T. C. Terwilliger, P. H. Zwart, *Acta Crystallogr. D Biol. Crystallogr.* **2010**, 213-221.
11. P. Emsley, B. Lohkamp, W. G. Scott, K. Cowtan, PHENIX: a comprehensive Python-based system for macromolecular structure solution, *Acta Crystallogr. D Biol. Crystallogr.* **2010**, 486-501.
12. A. A. Vaguine, J. Richelle, S. J. Wodak, SFCHECK: a unified set of procedures for evaluating the quality of macromolecular structure-factor data and their agreement with the atomic model, *Acta Crystallogr. D Biol. Crystallogr.* **1999**, *55*, 191-205.
13. Y. Liu, G. Bineva-Todd, R. W. Meek et al., A Bioorthogonal Precision Tool for Human N-Acetylglucosaminyltransferase V, *J. Am. Chem. Soc.* **2024**, *146*, 26707–26718.
14. A. Cioce, B. Calle, T. Rizou et al., Cell-specific bioorthogonal tagging of glycoproteins, *Nat. Commun.* **2022**, *13*, 6217.
15. A. Aatkar, A. Vuorinen, O. E. Longfield et al., Efficient Ligand Discovery Using Sulfur(VI) Fluoride Reactive Fragments, *ACS Chem. Biol.* **2023**, *18*, 1926-1937.

16. M. A. C. Neves, M. Totrov, R. Abagyan, Docking and scoring with ICM: the benchmarking results and strategies for improvement, *J. Comput. Aided Mol. Des.* **2012**, *26*, 675–686.
17. Y. A. Arnautova, A. Jagielska, H. A. Scheraga, A new force field (ECEPP-05) for peptides, proteins, and organic molecules, *J. Phys. Chem. B* **2006**, *110*, 5025–5044.
18. R. Abagyan, M. Totrov, Biased probability Monte Carlo conformational searches and electrostatic calculations for peptides and proteins, *J. Mol. Biol.* **1994**, *235*, 983–1002.
19. T. A. Halgren, Merck molecular force field. I. Basis, form, scope, parameterization, and performance of MMFF94, *J. Comput. Chem.* **1996**, *17*, 490–519.
